# Ren-Shen-Bu-Qi decoction alleviates exercise fatigue through activating PI3K/AKT/Nrf2 pathway in mice

**DOI:** 10.1186/s13020-024-01027-4

**Published:** 2024-11-05

**Authors:** Yangyang Chen, Tinghui Gao, Jing Bai, Wenjing Zhang, Yutong Zhou, Ruichang Zhao, Youhui Deng, Xiaogang Liu, Zhangjun Huang, Songtao Wang, Caihong Shen, Sijing Liu, Jinlin Guo

**Affiliations:** 1 State Key Laboratory of Southwestern Chinese Medicine Resources, College of Pharmacy, Chengdu University of Traditional Chinese Medicine, Chengdu, 611137 China; 2 College of Medical Technology, Chengdu University of Traditional Chinese Medicine, Chengdu, 611137 China; 3 Luzhou Laojiao Group Co. Ltd., Luzhou, People’s Republic of China; 4 National Engineering Research Center of Solid-State Brewing, Luzhou, People’s Republic of China

**Keywords:** Traditional Chinese medicine, Fatigue, Chemical composition, PI3K/AKT/Nrf2 signaling pathway

## Abstract

**Background:**

Fatigue is a prevalent issue that can lead individuals to a sub-health condition, impacting their work efficiency and quality of life. There are limited effective treatment options available for fatigue. Ren-Shen-Bu-Qi decoction (RSBQD) is a proprietary herbal remedy that is designed to address fatigue. However, the specific pharmacological mechanisms and basis of RSBQD are not yet fully understood.

**Purpose:**

This study aimed to investigate the pharmacological effects and mechanisms of RSBQD in a mouse model of exercise fatigue.

**Materials and methods:**

UPLC-Q-Orbitrap HRMS was used to analyze the chemical composition of RSBQD. The pharmacological basis and molecular mechanism of RSBQD on exercise fatigue were predicted using network pharmacology analysis. Subsequently, an exercise fatigue mouse model was established and used to analysis the effects of RSBQD. The potential mechanisms were verified by hematoxylin–eosin (HE) staining, real-time fluorescence quantitative PCR (RT-qPCR), Western blot (WB) and molecular docking.

**Results:**

The results showed that 88 main components of RSBQD were identified, which have mainly belonged to flavonoids and carboxylic acid compounds. The network pharmacology analysis indicated that RSBQD ameliorate fatigue through PI3K/AKT signaling pathway. Notably, RSBQD prolonged the swimming time and diminished body weight loss of exercise fatigue mice (*P* < 0.05). Meanwhile, RSBQD significantly alleviated the injury of liver and kidney induced by exhaustive exercise, and decreasing the serum alanine aminotransferase (ALT), aspartate aminotransferase (AST), urea and BUN levels (*P* < 0.05). In addition, RSBQD was found could relieve exercise fatigue by decreasing the content of creatine kinase (CK), lactate dehydrogenase (LDH), and lactic acid (LA), but increasing the blood glucose (GLU) and liver glycogen (HG) levels (*P* < 0.05). RSBQD also significantly increased the hepatic superoxide dismutase (SOD), glutathione peroxidase (GSH-Px) but decreased hepatic malondialdehyde (MDA) levels. Moreover, RSBQD was able to upregulate protein level of activated Nrf2 and PI3K/AKT signaling pathways.

**Conclusions:**

RSBQD mitigates exercise fatigue by reversing metabolic changes and reducing oxidative damage through the PI3K/AKT/Nrf2 signaling pathway. This study offers pharmacological support for the utilization of RSBQD in exercise fatigue treatment.

**Graphical Abstract:**

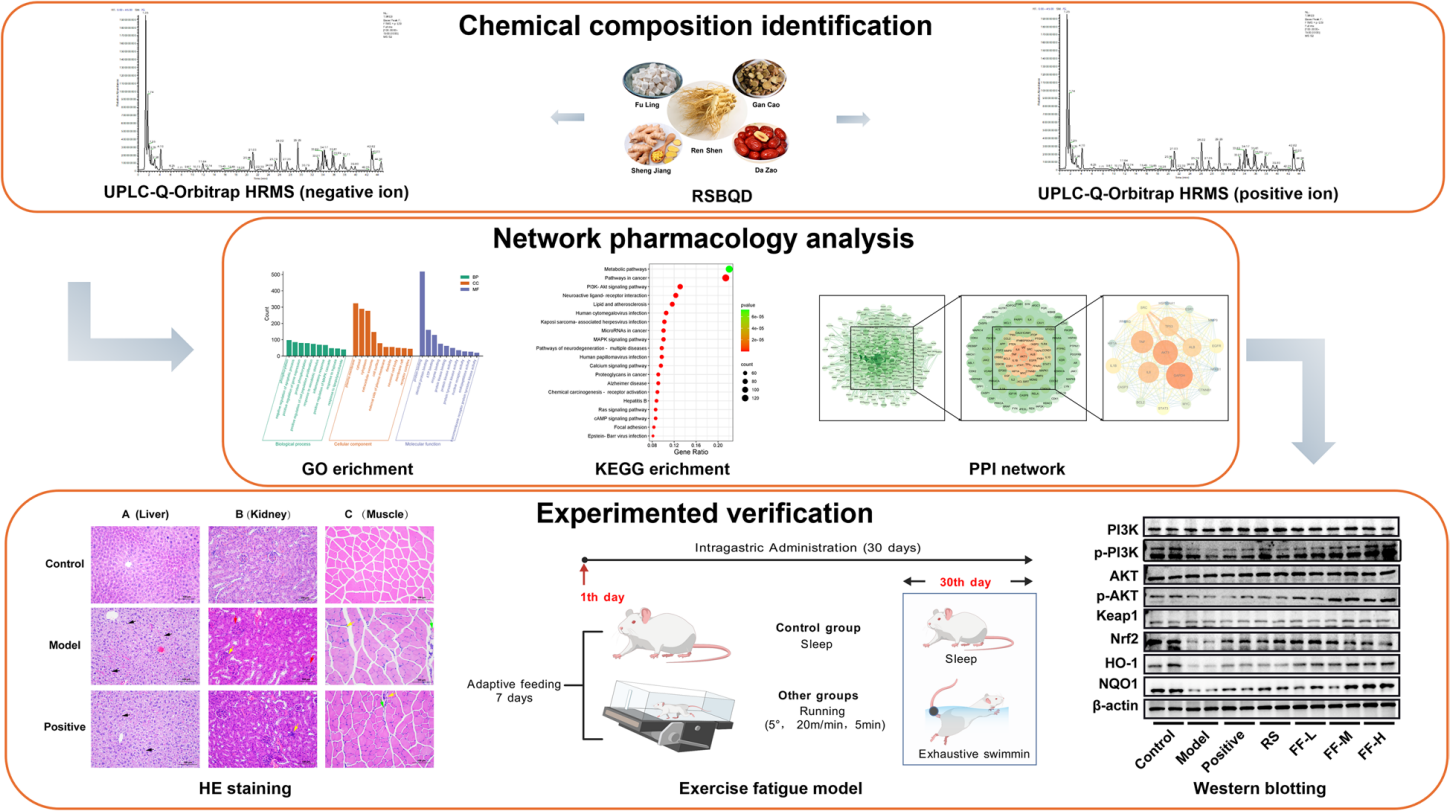

**Supplementary Information:**

The online version contains supplementary material available at 10.1186/s13020-024-01027-4.

## Introduction

Fatigue is a common symptom of suboptimal health, resulting in various physiological changes such as sleep disturbances, hormonal imbalances, metabolic issues, and weakened immune function [1, 2]. If left unaddressed, prolonged fatigue can progress to chronic fatigue syndrome, potentially leading to conditions like aging, anxiety, depression, cancer, and Parkinson’s disease [1–3]. Studies show that nearly 3% of the global adult population suffers from chronic fatigue lasting six months or longer [3]. Fatigue can be categorized into various types based on its manifestations and causes, of which exercise fatigue, as a specific form of physical fatigue, is of significant research value. Current fatigue management strategies mainly involve the use of medications such as corticosteroids, immunostimulants, antidepressants, sedatives, and antihistamines, which offer short-term relief by stimulating the brain and combating drowsiness [3–5]. However, prolonged use of these drugs may not be effective in halting fatigue progression, as some traditional stimulant medications have transient effects, addictive properties, and potential side effects [4, 5]. Therefore, there is a need to explore potential anti-exercise fatigue medications that demonstrate clear efficacy and minimal side effects.

Traditional Chinese Medicine (TCM) has attracted significant attention recently, as its great effectiveness and less side effects [6]. Based on the TCM theory, qi deficiency and spleen deficiency are the main cause of fatigue, therefore, qi and spleen deficiencies types of fatigue account for a larger proportion of all fatigue types [7, 8]. Ren-Shen-Bu-Qi decoration (RSBQD) is a proprietary herbal remedy of hospital of Chengdu University of TCM indicated for qi and spleen deficiency. It consists of 5 medicine food homology plant, including Ren Shen (the root of *Panax ginseng* C. A. Mey., Ginseng), Fu Ling (the root of *Wolfiporia cocos* (F.A. Wolf) Ryvarden & Gilb.), Da Zao (the fruits of *Ziziphus jujuba* Mill.), Sheng Jiang (the root of *Zingiber officinale* Rosc.) and Gan Cao (the root of *Glycyrrhiza uralensis* Fisch.,). Ginseng was identified as the primary herb in TCM and was first documented in the “*Shennong Ben Cao Jing*” (About 200 A.D.). It is commonly utilized as a tonic to promote longevity, boost energy levels, and enhance overall well-being. Ginseng has historically been employed to combat fatigue, as noted in various ancient texts such as the “*Shennong Ben Cao Jing*” (About 200 A.D.), “*Ren Zhai Zhi Zhi Fang Lun*” (1264 A.D.) and the “*Jiangxue Dan Shu*” in Ming Dynasty. In the RSBQD, ginseng is utilized to replenish qi, Fu Ling is employed to fortify the spleen and address dampness, Da Zhao is used to enhance qi, thereby amplifying the qi-boosting properties of ginseng. Sheng Jiang is incorporated to reinforce the spleen and dispel dampness, while Gan Cao is included to further support qi and harmonize the combination of herbs. Therefore, RSBQD is applied in clinical settings to alleviate fatigue associated with Qi-deficiency and spleen-deficiency. In the “*Huangdi Neijing*” (circa third-second century BCE), it is documented that “exertion depletes qi”, meaning that physical labor or exercise can lead to a deficiency of yang qi in the body, resulting in symptoms of physical fatigue such as limb weakness, general tiredness, and lassitude, which are consistent with the concept of exercise-induced fatigue in modern medicine. Therefore, RSBQD can also be used for the treatment of exercise-induced fatigue. However, the specific pharmacological mechanisms and basis of RSBQD are not yet fully understood.

Numerous theories have been proposed to explain the mechanisms of exercise fatigue, including the radical theory [9–11]. This theory posits that the buildup of reactive free radicals leads to oxidative stress, which in turn induces physical fatigue during excessive exercise [9–11]. As a result, antioxidants that modulate reactive oxygen species are considered essential in combating exercise fatigue. Nuclear factor erythroid 2-related factor-2 (Nrf2) is a crucial marker of antioxidant signaling, regulating the expression of downstream detoxification enzymes and antioxidants such as heme oxygenase 1 (HO-1) and NAD(P)H quinone oxidoreductase 1 (NQO1) [9–14]. Nrf2 activity is negatively controlled by Kelch-like ECH-associated protein 1 (Keap1), which also plays a role in the HO-1-mediated antioxidant response [9–11]. HO-1 could protected cells from oxidizing agents by converting heme into biologically active agents [9–11]. Numerous studies have highlighted the protective role of the Nrf2/HO-1 pathway against exercise fatigue [10, 11]. Additionally, several studies have shown that Nrf2 can be modulated by the phosphoinositide 3-kinase (PI3K)/protein kinase B (Akt) signaling pathway to play a protective role in exercise fatigue [15, 16]. Therefore, activation of the PI3K/Akt/Nrf2 pathway may be an important mechanism to alleviate exercise fatigue.

In this study, we utilized UPLC-Q-Orbitrap HRMS to identify the main active compounds in RSBQD and predicted the key targets and signaling pathways involved in alleviating exercise fatigue by network pharmacology analysis. Subsequently, an exercise fatigue mouse model was established, and RT-qPCR, Western blot and molecular docking analysis were used to confirm the anti-exercise fatigue mechanism. This integrated multidisciplinary methodology enhances our understanding of how RSBQD combats exercise fatigue and offers robust evidence for the advancement and utilization of natural remedies.

## Materials and methods

### RSBQD and Ren Shen preparation

RSBQD and Ren Shen (RS) powder were provided by Shanghai Tianyuan Botanical Products Co. RSBQD is the extracts of Ren Shen (the root of *Panax ginseng* C. A. Mey.), Fu Ling (the root of *Wolfiporia cocos* (F.A. Wolf) Ryvarden & Gilb.), Da Zao (the fruits of *Ziziphus jujuba* Mill.), Sheng Jiang (the root of *Zingiber officinale* Rosc.) and Gan Cao (the root of *Glycyrrhiza uralensis* Fisch.,) in a ratio of 3:3:3:2:2. RS extract is derived from the root of *Panax ginseng* C. A. Mey., and the extraction method is consistent with RSBQD. The powder was diluted into sterile water before use.

### Reagents

Vitamin C (M3121) was purchased from Abmole Bioscience Inc. (Shanghai, China). The standards of serum AST (105–000443-00), ALT (105–000442-00), CK (105–000458-00), LDH (105–000446-00), and UREA (105–000452-00) were obtained from Mindary (ShenZhen, China). The glycogen (M1510A), BUN (M1605A), MDA (M0106A) and GLU (M0506A) detection kit were purchased from Yanxi Bio Co. (Suzhou, China). The SOD (A001-3–2) and GLU (F006-1–1) detection kit was from Nanjing Jiancheng Bioengineering Institute (Nanjing, China) and ROBIO (Shanghai, China), respectively. The LA (E-BC-K044-M) and pyruvate kinase (PK, E-BC-K611-M) kit were obtained from Elabscience (Wuhan, China). The primary antibodies of PI3K (AF7921), p-PI3K (AF3242), AKT (AF6260), p-AKT (AF6261), Keap1(AF5266) and HO-1(AF5393) were purchased from Affinity (Jiangsu, China). The primary antibodie of NQO1 (PB0526) was obtained from Boster (Wuhan, China). The primary antibodie of β-actin (66,009–1-1g) was purchased from Proteintech (Wuhan, China).

### UPLC-Q-Orbitrap HRMS analysis

As decribed previously [17], the chemical composition of RSBQD was analyzed using a Vanquish type UPLC-Q-Orbitrap HRMS and a Thermo Scientific Accucore™ C_18_ column (3 mm × 100 mm, 2.6 μm). Details of the mobile phase and analytical conditions can be found in the supplementary materials. Data analysis was conducted with Compound Discoverer 3.0, utilizing a local high-resolution database of Chinese herbal medicine components and the mzCloud network database. Matching parameters included a peak area threshold of 80,000, a mass deviation of less than 5 ppm for primary quasi-molecular ions and secondary fragments, and a match score above 80. For further compound identification, additional analyses were carried out with Xcalibur, Mass Frontier software, validation controls, literature references, and databases like Pubchem, HMDB, and MassBank.

### Network pharmacology analysis

As decribed previously [18], the targets of chemicals in RSBQD were screened using the Swiss Targets Prediction database and Traditional Chinese Medicine Systems Pharmacology Database and Analysis Platform. Data on “Exercise fatigue” targets were retrieved from the GeneCards, DrugBank, and Online Mendelian Inheritance in Man database. The intersecting targets were selected and used to analyze the protein and protein interaction (PPI) networks using the STRING 12.0 database and displayed with Cytoscape 3.7.2 software. These overlapping targets were used for Gene Ontology (GO) and Kyoto Encyclopedia of Genes and Genomes (KEGG) emrichment analysis tyhrough the DAVID database, and the results were plotted and shown using the web tool bioinformatics.

### Animal treatment

All animal experiments received approval from the Ethics Committee for the Care and Use of Laboratory Animals at Chengdu University of Traditional Chinese Medicine (Approval No. 2024048).

Male ICR mice (6 weeks old, SPF) were purchased from SPF Biotechnology Co., Ltd (SCXK(Jing) 2019–0010, Beijing, China). After adaptive period, mice were divided into 7 groups (n = 10), the Control group (Distilled water, 500μL/kg body weight), the Model group (Distilled water, 500μL/kg body weight), the positive drug group (Vitamin C, 100 mg/kg body weight), the Ren Shen control (RS, 0.51 g/kg/day), the low dose (FF-L, 0.45 g/kg body weight), middle dose (FF-M, 0.9 g/kg body weight) and high dose (FF-H, 1.8 g/kg body weight) of RSBQD. Each mouse was gavaged once a day (10 mL/kg). The mid-dosage of RSBQD in mice is equivalent to 100 mg/kg powder (39 g herbs) for an adult per day, calculated as the equivalent dose ratio converted from body surface area. The calculation ensures that the administered dose is both relevant and safe for the experimental subjects. The dosage of 39 g of RSBQD is per day for an adult is derived from TCM practices, where such formulations are commonly used in clinical settings. The dosage of Ren Shen was consistent with the middle dose in the RSBQD. The experiment was conducted for 30 days, which was chosen to mimic the typical duration and dosage of herbal treatments used in TCM for alleviating chronic conditions such as fatigue. This extended period allows for the assessment of long-term effects of RSBQD. The clinical significance lies in evaluating the therapeutic potential and safety of RSBQD over a sustained period, providing insights into its efficacy and possible side effects. Excepted the Control group, all mice were subjected to daily forced running sessions at 25 m/min on a 5° incline for 5 min, continued over a period of 30 days to induce a sustained state of exercise fatigue. The modeling conditions were determined based on a pre-experiment, the details of which are presented in Table S3.

### Exhaustive swimming

As described previously [19], on day 30, 1 h after treatment, mice were subjected to a forced swimming test. Each mouse had a weight equivalent to 5% of its body mass attached to the base of its tail. The swim test was conducted in a tank measuring 50 cm × 45 cm × 40 cm, filled with water to a depth of 35 cm and maintained at 25 ± 1 °C. Exhaustion was determined when the mouse failed to keep its head above water for 8 consecutive seconds. The duration from the beginning of the test to the point of exhaustion was recorded.

### Biochemical test

After swimming, the samples of serum, liver, kidney, spleen and hindlimb muscle were collected. The serum ALT, AST, CK, LDH and Urea were detected by an automatic blood biochemistry analyzer (Mindray, ShenZhen, China). The serum GLU, LA, BUN, glycogen from the liver and muscle tissues, and the hepatic MDA, SOD and GPX were detected according to the protocols of these kits.

### HE staining

The collected tissues were fixed, dehydrated, embedded and cut into 3 μm pieces. These sections were stained with hematoxylin and eosin (HE) and pictured under a microscope (Nikon, Japan). The photographs were analyzed by two pathologists in a blinded fashion.

### RT-qPCR and WB analysis

The total RNA and protein of liver were extracted by TRIZOL and RIPA buffer, respectively. The RNA was reversed into cDNA and the mRNA levels were analyzed by RT-qPCR with primers in Table S2. The 2^−△△ct^ methods was used to normalize the mRNA levels with GAPDH as the reference. The protein was separated by SDS-PAGE and transferred into PVDF membranes. The membranes were then incubated with specific primary and secondary antibodies to detect the target protein levels, with β-actin serving as the loading control. The details of RT-qPCR and WB were described in supplementary methods.

### Molecular docking

The potential active ingredient structures were retrieved from Compound Discover software and saved in MOL format, which were then converted to SDF format. Concurrently, the structures of core target proteins were extracted from the RCSB PDB protein databases and stored in PDB format. Initially, molecular docking between the potential active ingredients and the core targets was performed using Maestro software. Subsequently, visualization of the docking outcomes was accomplished using PyMOL software. Ultimately, molecular docking scores were utilized to generate heat maps with Origin software.

### Statistical analysis

Data analysis was conducted with GraphPad Prism 9 (La Jolla, CA, USA). The results were evaluated using ANOVA with LSD test or the Kruskal–Wallis test. Results are presented as mean ± standard deviation (means ± SD), and a P value of less than 0.05 was considered statistically significant..

## Results

### Chemical composition of Ren-Shen-Bu-Qi decoction

**Fig. 1:**
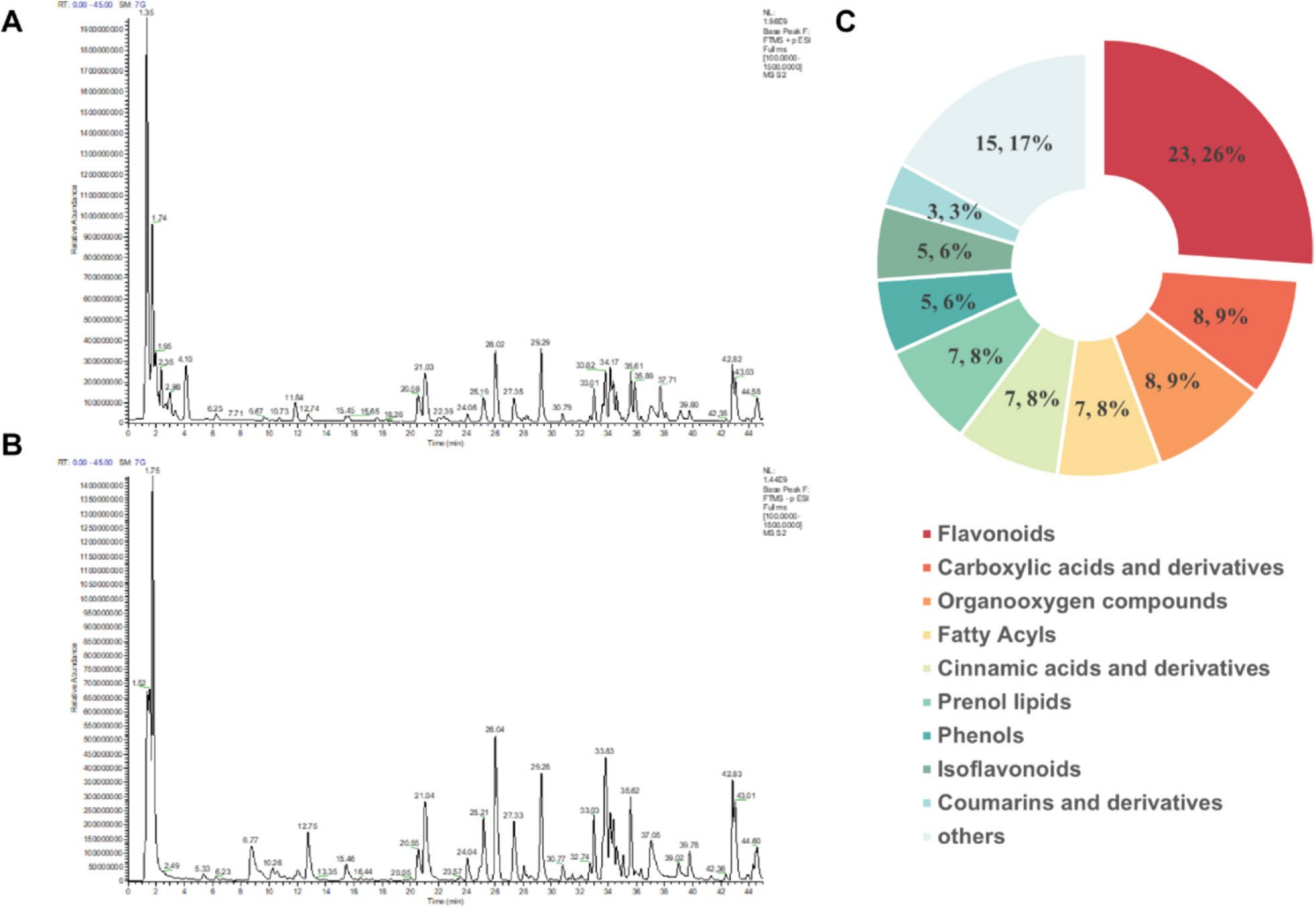
The UPLC-Q-Orbitrap HRMS analysis of RSBQD. **A**, **B** The RSBQD analyzed in positive ion mode (**A**) and negative ion mode (**B**). **C** Component classification diagram of RSBQD

The UPLC-Q-Orbitrap HRMS were used to analyze the chemical composition of RSBQD in both positive and negative ion mode (Fig. 1A, B). As shown in Table S2, 88 chemical components were identified from RSBQD. These compounds comprised of 23 flavonoids, 8 carboxylic acids and their derivatives, 8 organic oxides, 7 fatty acids, 7 cinnamic acids and their derivatives, 7 propargyl alcohol lipids, 5 phenols, 5 isoflavonoids, 3 coumarins and their derivatives, and 15 other substances (Fig. 1C). Among them, isoliquiritigenin boasts the highest relative content (Table 1).

**Table 1:** The top 15 of chemical components in RSBQD based on relative contents

NO	Compounds	Formula	m/z	Reference ion	RT [min]	Area
1	Isoliquiritigenin	C_15_ H_12_ O_4_	257.08	[M + H]^+1^	21.03	4.39 × 10^9^
2	Naringin	C_27_ H_32_ O_14_	579.17	[M-H]^−1^	25.19	3.10 × 10^9^
3	20(R)-Ginsenoside Rg2	C_42_ H_72_ O_13_	829.50	[M + FA-H]^−1^	34.37	2.40 × 10^9^
4	DL-Norleucine	C_6_ H_13_ N O_2_	132.10	[M + H]^+1^	2.01	1.57 × 10^9^
5	Sinapine	C_16_ H_23_ N O_5_	310.16	[M + H]^+1^	11.84	1.56 × 10^9^
6	Hesperetin	C_16_ H_14_ O_6_	301.07	[M-H]^−1^	33.64	1.48 × 10^9^
7	3-O-Feruloylquinic acid	C_17_ H_20_ O_9_	367.10	[M-H]^−1^	15.45	1.25 × 10^9^
8	Guanosine	C_10_ H_13_ N_5_ O_5_	284.10	[M + H]^+1^	1.91	1.02 × 10^9^
9	Azelaic acid	C_9_ H_16_ O_4_	187.10	[M-H]^−1^	24.91	8.91 × 10^9^
10	trans-3-Indoleacrylic acid	C_11_ H_9_ N O_2_	188.07	[M + H]^+1^	6.24	8.19 × 10^9^
11	3-p-coumaroylquinic acid	C_16_ H_18_ O_8_	337.09	[M-H]^−1^	12.97	7.89 × 10^9^
12	Zapotin	C_19_ H_18_ O_6_	343.12	[M + H]^+1^	38.10	7.21 × 10^9^
13	Poncirin	C_28_ H_34_ O_14_	639.19	[M + FA-H]^−1^	30.78	6.15 × 10^9^
14	Kojic acid	C_6_ H_6_ O_4_	143.03	[M + H]^+1^	5.05	5.71 × 10^9^
15	Formononetin	C_16_ H_12_ O_4_	269.08	[M + H]^+1^	35.44	5.30 × 10^9^

### Network pharmacology analysis of Ren-Shen-Bu-Qi decoction

**Fig. 2:**
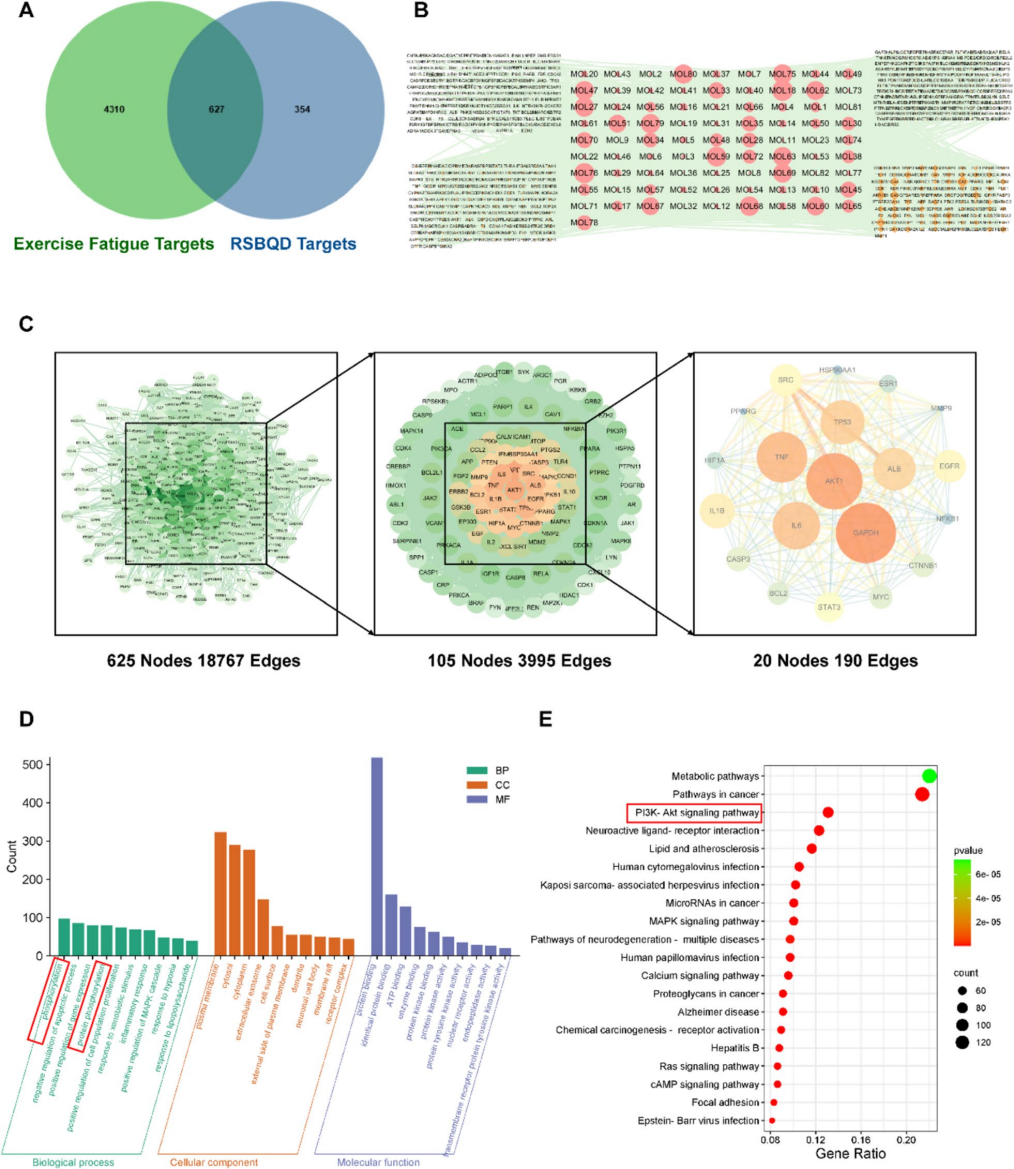
Prediction of anti-exercise fatigue targets and active ingredients of RSBQD using network pharmacology. **A** Venny analysis of RSBQD targets and exercise fatigue targets. **B** Component—potential targets network. **C** PPI protein interaction of potential targets. **D** GO enrichment analysis of potential targets. **E** KEGG enrichment analysis of potential targets

To potential mechanism of RSBQD on exercise fatigue, we collected the targets of RSBQD components and exercise fatigue for network pharmacology analysis. As shown in Fig. 2A, there are 627 overlapping genes between them. Filtering by maximal clique centrality rank, we got 20 key genes, and their PPI network were presented in Fig. 2C. Notably, these gene mainly mediated the biological processes of “phosphorylation” and “protein phosphorylation” (Fig. 2D). In addition, these genes are mainly enriched in the PI3K-AKT signaling pathway (Fig. 2E). Therefore, we speculated that phosphorylation of key proteins on the PI3K-AKT signaling pathway may play a key role in the anti-exercise fatigue effect of RSBQD. Moreover, the component-potential targets network indicated that sinapine, tangeritin, arctigenin, and zapotin may be the key components of RSBQD involved in the alleviation of exercise fatigue (Fig. 2B).

### Ren-Shen-Bu-Qi decoction improves the exercise performance and alleviates injury induced by exercise fatigue

**Fig. 3:**
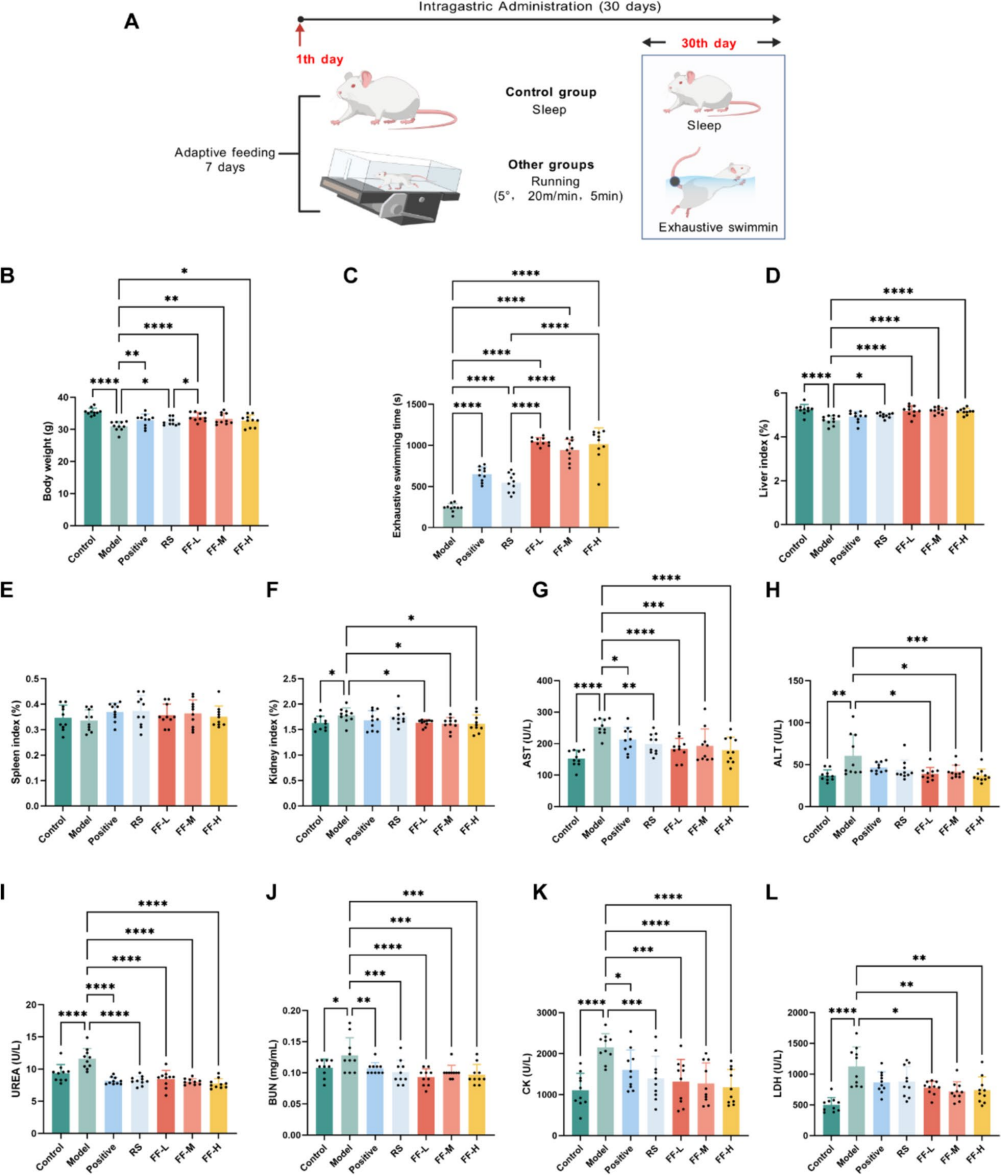
Effect of RSBQD improved the exercise performance and alleviates injury induced by exercise fatigue (n = 10). **A** Animal study design. **B** Body weight gain. **C** Exhaustive swimming time. **D**–**F** The liver (**D**), spleen (**E**) and kidney (**F**) index. **G**–**L** Serum AST (**G**), ALT (**H**), UREA (**I**), BUN (**J**), CK (**K**) and LDH (**L**) levels. *P < 0.05, **P < 0.01, ***P < 0.001, ****P < 0.0001

**Fig. 4:**
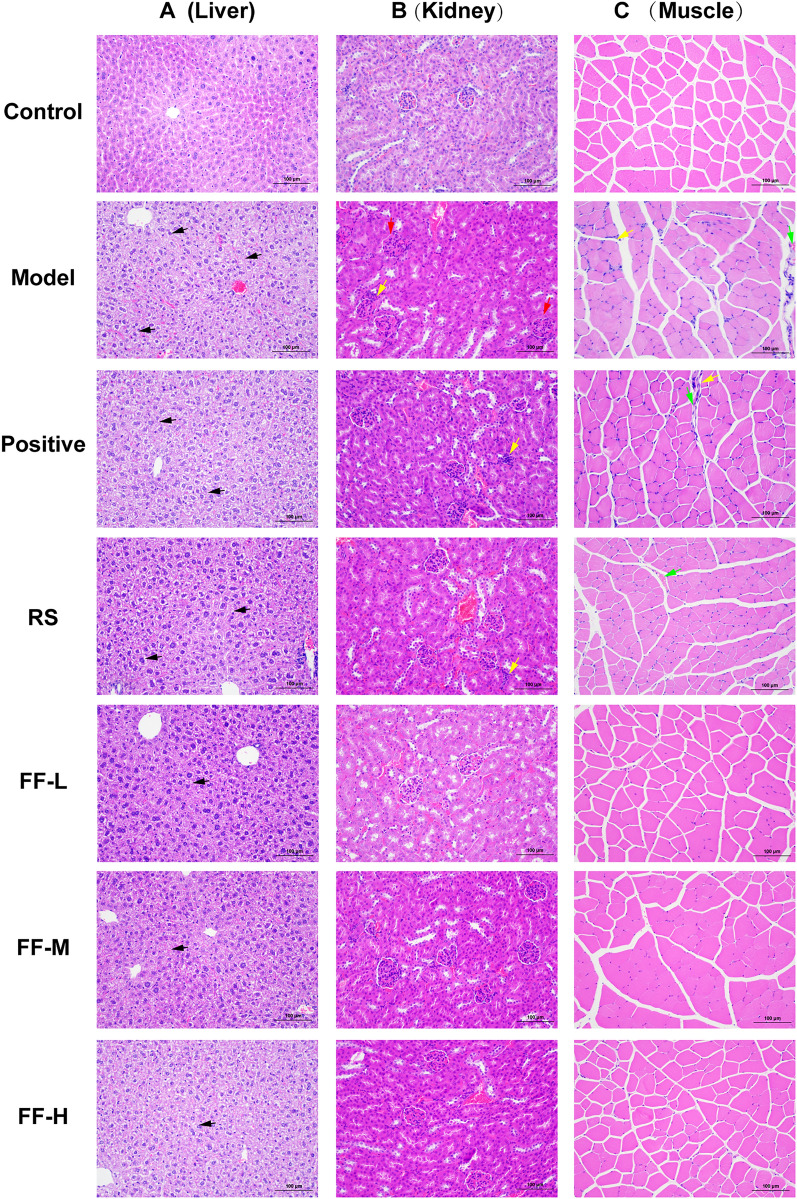
Effect of RSBQD on alleviating tissue lesions in exercise-fatigued mice (n = 10). **A** HE staining of liver (200 ×). **B** HE staining of kidney (200 ×). **C** HE staining of muscle (200 ×). Scale bar = 100 μm. The black arrow indicates hepatocyte degeneration and cytoplasmic lysis forming lumens with fatty vacuoles; the red arrow indicates narrowing of glomerular interstitial space; the yellow arrow indicates inflammatory cell infiltration; and the green arrow indicates vasodilation with hemorrhage

To confirm the anti-exercise fatigue properties of RSBQD, an exercise fatigue mouse model was created and administered RSBQD for a duration of one month (Fig. 3A). The body weight of the mice in each group was recorded at 7-day intervals throughout the experiment, revealing RSBQD reduces body weight loss in exercise fatigued mice (*P* < 0.05, Fig. 3B). Exhaustive swimming time is a classical index to evaluate the physical fatigue. As depicted in Fig. 3C, the swimming time of the model group was 241 ± 49.64 s. Following administration, mice in the positive, Ren Shen, and RSBQD groups showed a significantly prolonged time to exhaustion compared to those in the Model group (*P* < 0.0001, Fig. 3C). Notably, the low, middle, and high doses of RSBQD notably extended swimming time by 332%, 289%, and 319%, respectively. Although no significant dose-dependent trend was observed, all three doses demonstrated improved exercise performance compared to the Ren Shen control (*P* < 0.0001, Fig. 3C). Concomitantly, liver index was markedly increased, and the kidney index was decreased after RSBQD treatment, while spleen index did not differ significantly among the groups (*P* < 0.05, Fig. 3D–F), suggesting that RSBQD could reduce liver and kidney injuries caused by exercise fatigue. We therefore evaluate the liver, kidney, or muscle function-related index, such as serum AST, ALT, UREA, BUN, CK and LDH. As expected, the serum levels of these indices were significantly elevated in the model group mice compared to the control group (*P* < 0.05, Fig. 3G–L). While vitamin C and Ren Shen did not impact serum ALT and LDH levels, Ren Shen and RSBQD effectively prevented the gradual rise in these levels (*P* < 0.05, Fig. 3G–L). In addition, the administration of Ren Shen and RSBQD, as well as the positive control, helped reduce the elevated levels of UREA, BUN, CK, and LDH caused by exercise fatigue. These findings suggest that the excessive high intensity running training resulted in liver, kidney, and muscle damage in mice, which was mitigated by RSBQD. To validate these results, HE staining of the liver, kidney, and muscles was conducted. Figure 4A shows hepatocyte degeneration and cytoplasmic lysis forming lumens with fatty vacuoles (black arrow) in the model group, conditions that were significantly improved by RSBQD. In Fig. 4B, the model group exhibited a reduced glomerular balloon gap (red arrows) and mild inflammatory cell infiltration (yellow arrows); however, these lesions were absent in all RSBQD dose groups. Figure 4C depicts inflammatory cell infiltration (yellow arrows) and vasodilation with hemorrhage (green arrows) in the muscle tissues of the model group, which were mitigated to some extent by RSBQD. Taken together, these findings indicate that RSBQD enhances exercise endurance in exercise fatigued mice and alleviates liver, kidney, and muscle damage induced by overtraining.

### Ren-Shen-Bu-Qi decoction could relieve exercise fatigue by ameliorating energy metabolism

**Fig. 5:**
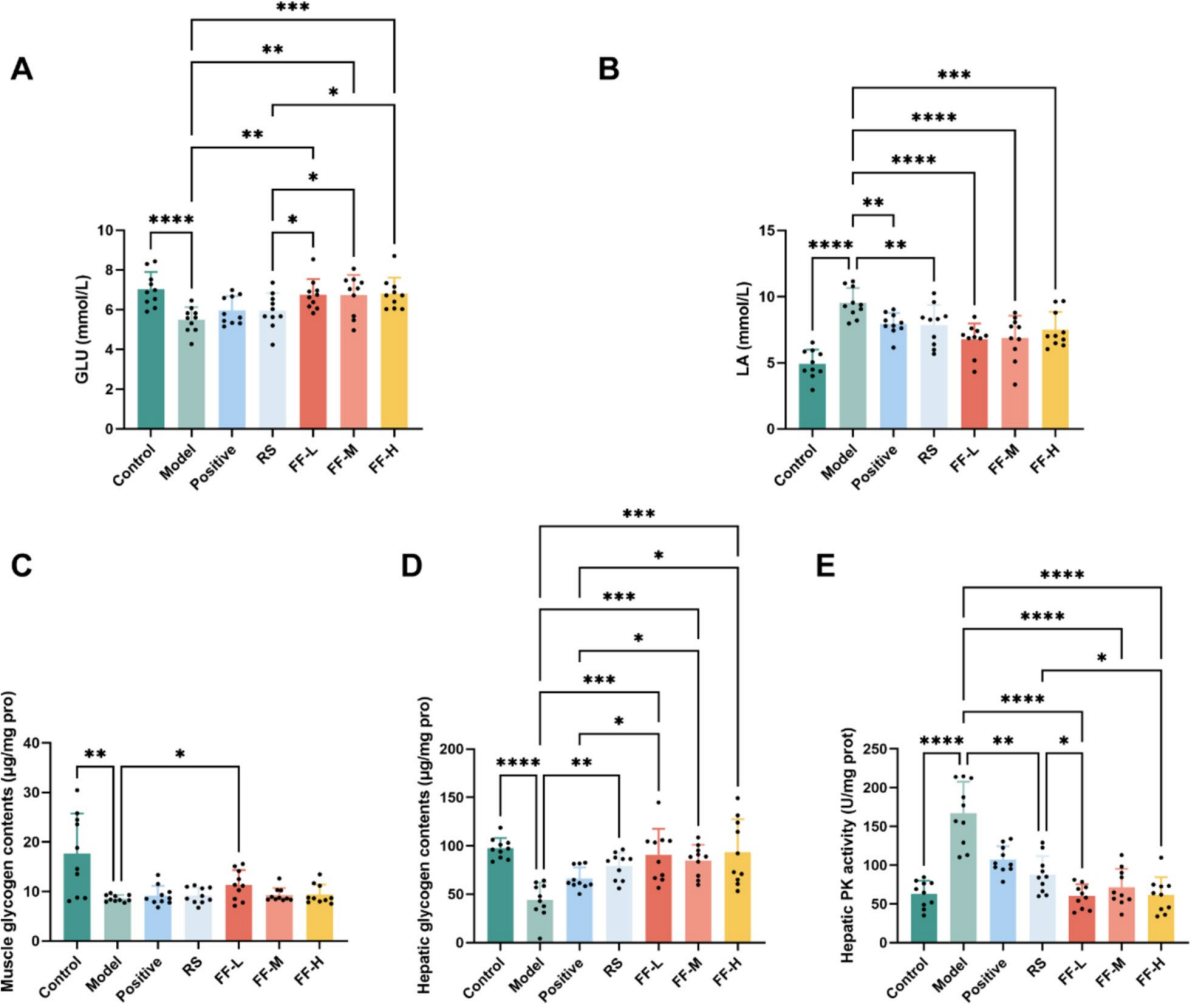
RSBQD ameliorated energy metabolism in exercise fatigued mice (n = 10). **A** Serum GLU contents. **B** Serum LA contents. **C** Muscle glycogen contents. **D** Hepatic glycogen contents. **E** Hepatic pyruvate kinase activity. *P < 0.05, **P < 0.01, ***P < 0.001, ****P < 0.0001

Exercise fatigue is commonly linked to abnormal energy metabolism. To explore the potential of RSBQD in alleviating exercise fatigue by modulating energy metabolism, we assessed serum levels of LA and GLU, as well as muscle glycogen, hepatic glycogen contents, and hepatic pyruvate kinase activity. Compared to the Control group, the Model group exhibited decreased GLU and increased LA levels (Fig. 5A and B, P < 0.001), indicating metabolic disorder and exercise fatigue in the mice. Importantly, RSBQD not only increased GLU levels but also decreased LA levels (Fig. 5A and B, P < 0.05), suggesting its efficacy in relieving exercise fatigue and restoring normal metabolism. Moreover, RSBQD showed superior effects in increasing GLU and decreasing LA compared to the positive drug control and Ren Shen. Additionally, myoglycogen and hepatic glycogen content decreased significantly in the Model group (Fig. 5C and D, P < 0.01), while PK activity increased (Fig. 5E, P < 0.0001), indicating energy overconsumption due to high intensity running training. Interestingly, only the low dose of RSBQD restored myo-glycogen content (Fig. 5C, P < 0.01), while all doses increased hepatic glycogen content (Fig. 5D, P < 0.01), outperforming the positive drug and Ren Shen. Specifically, hepatic glycogen levels increased by 105%, 92%, and 112% with the low, middle, and high doses of RSBQD intervention compared to the Model group (Fig. 5D, P < 0.001). In addition, RSBQD intervention reduced hepatic pyruvate kinase activity (Fig. 5E, P < 0.0001), surpassing the effect of Ren Shen. These findings suggest that RSBQD effectively enhances energy metabolism, particularly by promoting hepatic glycogen production in exercise fatigue mice.

### Ren-Shen-Bu-Qi decoction relieve exercise fatigue through PI3K/AKT/Nrf2 signaling pathway

**Fig. 6:**
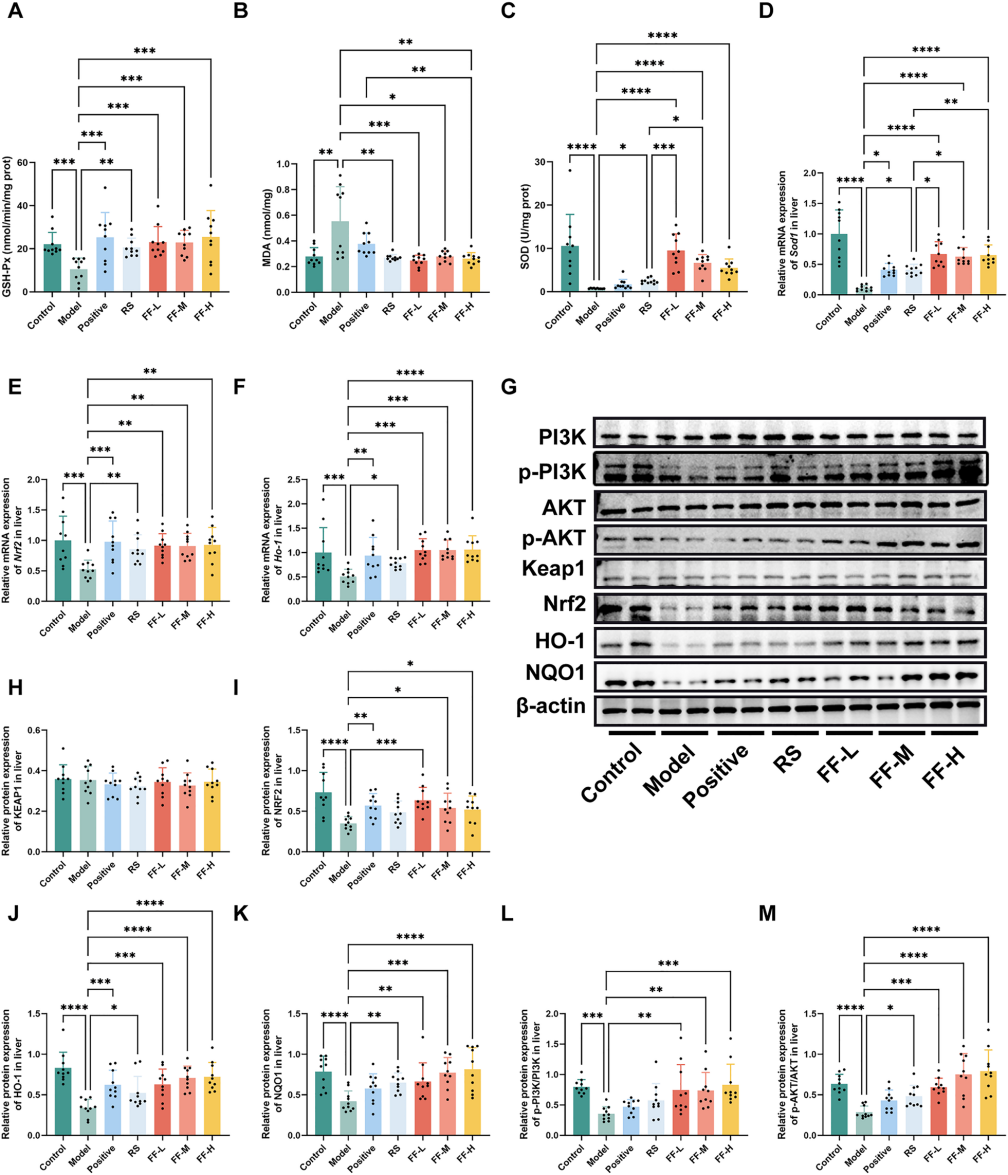
RSBQD activated PI3K/AKT/Nrf2 signaling pathway in exercise fatigued mice (n = 10). **A** Hepatic GSH-Px activity. **B** Hepatic SOD activity. **C** Hepatic MDA content. **D**–**F** The relative mRNA levels of *Sod1* (**D**), *Nrf2* (**E**), and *Ho-1* (**F**). **G** Representative images of Western blot. **H**–**K** Relative protein levels of KEAP1 (**H**), NRF2 (**I**), HO-1 (**J**), and NQO1 (**K**). (**L**, **M**) The ratio of p-PI3K/PI3K **L** and p-AKT/AKT **M**. *P < 0.05, **P < 0.01, ***P < 0.001, ****P < 0.0001

Excessive oxidative stress is a key factor in the development of exercise-induced fatigue, ultimately leading to decreased exercise performance [11, 15, 16]. MDA is commonly used as a biomarker for lipid peroxidation, while SOD and GSH-Px are key antioxidants [20]. In this study, we examined the levels and activity of GSH-Px, SOD, and MDA in the liver. As depicted in Fig. 6A–C, the Model group exhibited decreased GSH-Px and SOD activities, along with increased MDA content (*P* < 0.01). This suggested that continuous high intensity running training induced oxidative stress in mice, leading to oxidative damage in hepatocytes. Additionally, the groups treated with Ren Shen and various doses of RSBQD showed enhanced GSH-Px and SOD activity, as well as reduced MDA content (*P* < 0.05). Interestingly, while the positive drug significantly regulated the GSH-Px activity and MDA content, it did not affect the SOD activity (Fig. 6A–C). Specifically, the hepatic SOD enzyme activities in the low, middle, and high doses of RSBQD groups were significantly higher than those in the Model group by 1191%, 802%, and 649%, respectively (Fig. 6B, P < 0.0001). Furthermore, the hepatic SOD activities in these groups surpassed those in the Ren Shen group (Fig. 6C, P < 0.05). This was supported by RT-qPCR results, which indicated higher hepatic SOD1 mRNA expression levels in the RSBQD group compared to the Model and Ren Shen groups (Fig. 6C, P < 0.05).

Nrf2 serves as a key regulator in the management of oxidative stress by activating transcription, leading to enhanced secretion of antioxidant enzymes and regulation of antioxidant gene expressions, including downstream genes such as HO-1 and NQO1[12, 13]. Moreover, the Nrf2 signaling pathway were greatly regulated by the activation of the PI3K/Akt [21]. Therefore, to explore the mechanism by which RSBQD impacts liver function, we analyzed the expression of Keap1, Nrf2, and HO-1 genes and proteins in the liver using RT-qPCR and WB, respectively. We also examined changes in PI3K/AKT signaling pathway proteins, such as p-PI3K, PI3K, p-AKT, and AKT. As shown in Fig. 6E–K, it revealed that RSBQD did not affect Keap1 expression but significantly increased the expression of Nrf2, HO-1, and NQO1 (*P* < 0.01). Furthermore, RSBQD notably elevated the ratios of p-PI3K/PI3K and p-AKT/AKT (Fig. 6G, L–M Fig. S1, *P* < 0.01), indicating that RSBQD promotes the phosphorylation of the PI3K/AKT signaling pathway. Based on the comprehensive analysis of the above experimental results, it can be seen that the phosphorylation of PI3K and AKT is promoted by RSBQD, leading to the activation of Nrf2. The activated Nrf2 is then translocated to the cell nucleus, where it induces the expression of downstream antioxidant enzymes, such as HO-1 and NQO1, thereby enhancing the cell's antioxidant capacity and alleviating oxidative stress.

### Molecular docking

**Fig. 7:**
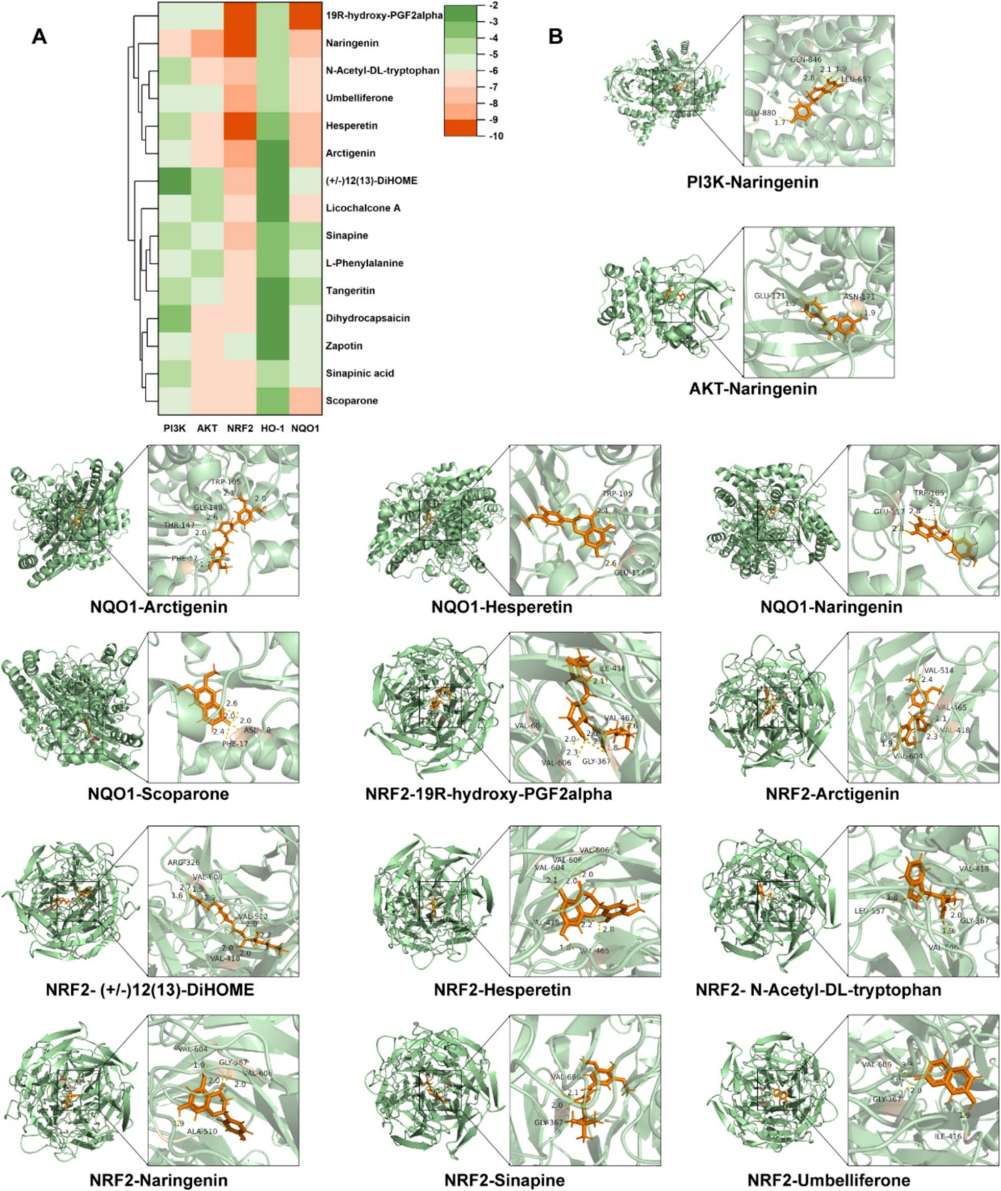
RSBQD activated PI3K/AKT/Nrf2 signaling pathway in exercise fatigue. **A** Heatmap of component-target protein docking scores. **B** Visualization of docking results between representative compounds and target protein molecules

To further investigate the anti-exercise fatigue active constituents within RSBQD, we conducted molecular docking of the top 15 active ingredients ranked by degree values in the Component—potential targets network of Sect. "Network pharmacology analysis of Ren-Shen-Bu-Qi decoction" with PI3K, AKT, NRF2, HO-1, and NQO1 proteins. As illustrated in Fig. 7A, B, most predicted active ingredients could form stable complexes (the score <  − 6) with AKT, NRF2, and NQO1, notably Naringenin. However, PI3K and HO-1 exhibited limited capacity to form stable complexes with the active ingredients. These findings suggest that Naringenin might be a key active constituent in RSBQD exerting anti-exercise fatigue effects through the PI3K/AKT/NRF2 pathway. Furthermore, it further substantiates that RSBQD regulates HO-1 not through direct interaction but acts upstream of HO-1, indirectly increasing its expression levels to exert antioxidant stress effects.

## Discussion

Exercise fatigue is a common symptom in modern society, often associated with irregular exercise habits, sedentary lifestyles, increased workloads, and mental stress. [1, 2]. There is growing interest in using TCM to address these symptoms [3, 6, 7]. Ren-Shen-Bu-Qi Decoction (RSBQD) is a proprietary herbal remedy in Chengdu University of Traditional Chinese Medicine, proposed to alleviate fatigue. This study systematically analyzed the chemical composition of RSBQD and investigated its potential in alleviating exercise-induced fatigue using network pharmacology and an exercise fatigue mouse model.

Our study is the first to comprehensively analyze the chemical composition of RSBQD using UPLC-Q-Orbitrap HRMS and identify its key compounds such as Sinapine, Tangeritin, Arctigenin, and Zapotin. These compounds have been shown to possess significant antioxidant and anti-inflammatory properties in existing literature [22, 23]. While the PI3K/AKT and Nrf2 signaling pathways have been studied in the context of exercise-induced fatigue [24, 25], the innovation of this study lies in the systematic evaluation of the synergistic effects of the multiple components of RSBQD in alleviating exercise-induced fatigue. These findings highlight the potential of RSBQD as a natural therapeutic, particularly in multi-component, multi-target treatment strategies.

Compared to other studies, our research not only confirms the anti-exercise fatigue effects of Ren Shen but also reveals the synergistic effects of multiple components within RSBQD. Previous studies have shown that Ren Shen and its major components, ginsenosides, significantly enhance physical strength, combat fatigue, and boost immunity [26, 27]. However, our study found that the anti-exercise fatigue effects of RSBQD were significantly superior to those of ginseng alone, likely due to the “matching-synergy” principle of Traditional Chinese Medicine, where the combined action of various components enhances therapeutic efficacy. Moreover, when compared to Vitamin C, which is widely recognized for its antioxidant properties and ability to alleviate fatigue, RSBQD demonstrated superior efficacy. While Vitamin C primarily functions by reducing oxidative stress, RSBQD not only enhances antioxidant capacity but also improves energy metabolism, protects liver and kidney function, and reduces inflammation. This multi-targeted approach makes RSBQD a more comprehensive solution for fatigue management. Additionally, RSBQD, as a medicinal food homology prescription, offers a higher degree of safety and compliance, making it a practical and sustainable option for long-term fatigue management. Our experimental results demonstrated that RSBQD significantly extended swimming time, reduced serum AST and ALT levels, and provided better liver protection compared to Vitamin C and Ren Shen alone.

This study shows that RSBQD significantly enhances antioxidant capacity and hepatic energy metabolism through the synergistic action of multiple signaling pathways. The PI3K/AKT pathway plays a crucial role in cellular metabolism, proliferation, and survival, with its activation promoting glycogen synthesis and fatty acid oxidation, thereby improving energy metabolism [28, 29]. Existing research indicates that the PI3K/AKT pathway plays an important role in alleviating exercise-induced fatigue by regulating energy metabolism and reducing oxidative stress [15, 16]. The Nrf2 pathway is a classic antioxidant and anti-stress signaling pathway that helps reduce oxidative stress and inflammation, protecting cells from damage [9–11]. This study is the first to show that RSBQD, through the synergistic action of these two pathways, not only improves serum glucose levels but also significantly increases hepatic glycogen reserves, indicating its important role in restoring energy balance and improving exercise fatigue.

Our findings suggest that RSBQD has the potential to be an effective natural therapeutic for managing and treating exercise-induced fatigue. Its multi-component, multi-target characteristics provide unique advantages in combating fatigue, especially for individuals engaged in prolonged high-intensity physical activities or those suffering from chronic fatigue syndrome. RSBQD not only demonstrated superior effects in prolonging exercise duration and reducing exercise-induced biochemical abnormalities but also showed significant protective effects on the liver, kidneys, and muscle tissues (Fig. 3, 4). These findings provide strong support for the clinical application of RSBQD and pave the way for its further development in athlete recovery and daily fatigue management for the general population.

Despite the significant progress made in this study, there are several limitations. Firstly, our research mainly focused on an animal model of exercise-induced fatigue, and further human clinical trials are needed to verify the efficacy and safety of RSBQD. Secondly, the specific mechanisms of action of each component within RSBQD were not fully elucidated. Future studies should further explore the individual and combined effects of these components. Additionally, the impact of RSBQD on mental fatigue remains unclear, which is an important direction for future research.

## Conclusion

**Fig. 8:**
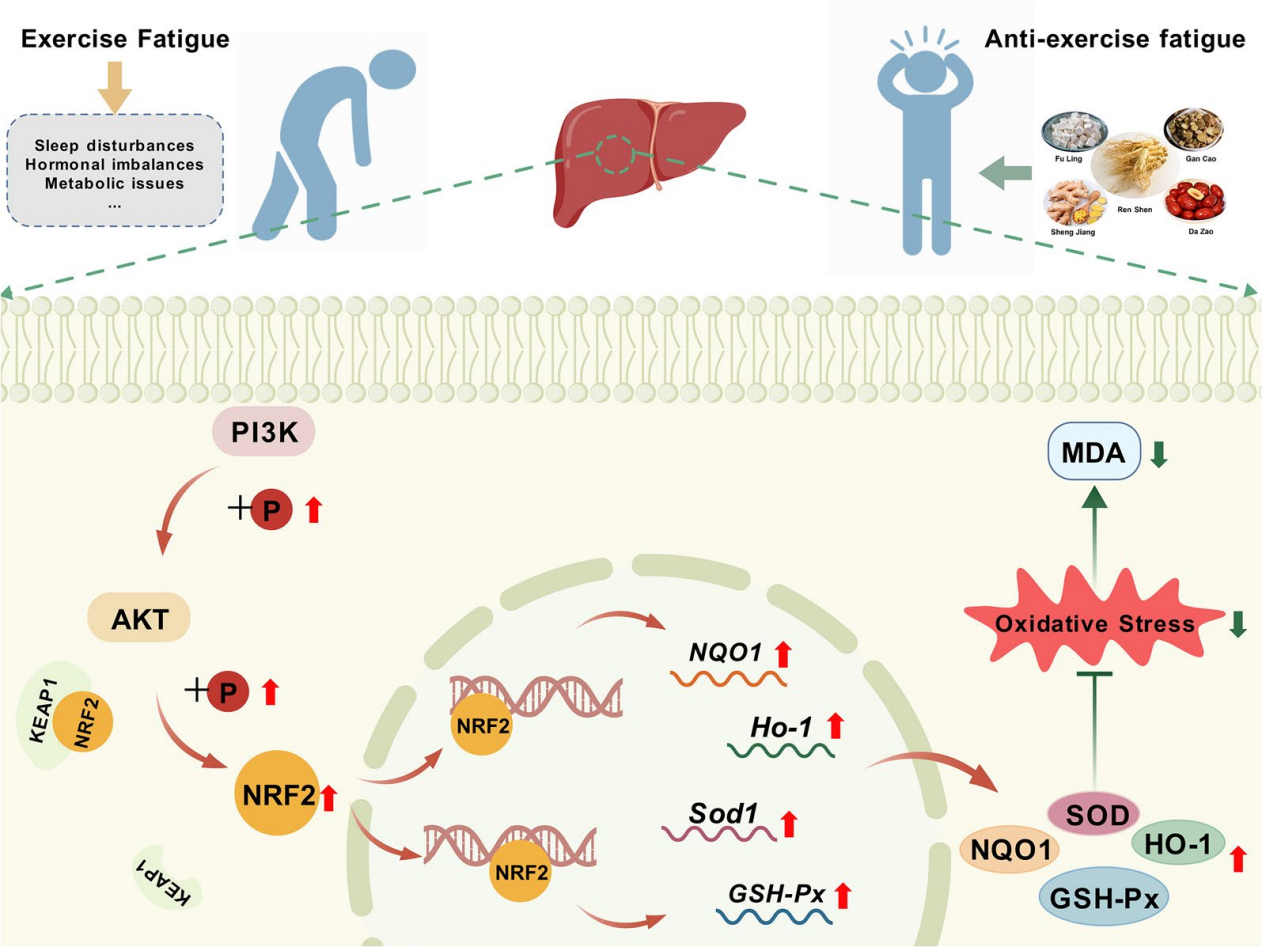
The potential mechanism of RSBQD on exercise fatigue. (Created with gdp.renlab.cn)

In this study, we have identified 88 components in RSBQD and explored its potential mechanism in alleviating exercise fatigue through UPLC-Q-Orbitrap HRMS and network pharmacology analysis. Our findings suggested that RSBQD may relieve exercise fatigue and reduce injuries in the liver, kidney, and muscle in a mice model. This effect is likely mediated by regulating metabolism and reducing oxidative damage through the activation of the PI3K/AKT/Nrf2 signaling pathway (Fig. 8). This study provides substantial evidence supporting the efficacy of RSBQD in alleviating exercise-induced fatigue through its multi-component, multi-target approach, highlighting its clinical significance and potential for future therapeutic development.

## Supplementary Information

Supplementary file 1.

Supplementary file 2.

Supplementary file 3.

Supplementary file 4.

Supplementary file 5.

## Data Availability

All the data used to support the findings of this study are available from the corresponding author upon reasonable request.

## Abbreviations

RSBQD: Ren-Shen-Bu-Qi decoction
HE: Hematoxylin–eosin
RT-qPCR: Real-time fluorescence quantitative PCR
WB: Western blot
ALT: Aminotransferase
AST: Aspartate aminotransferase
CK: Creatine kinase
LDH: Lactate dehydrogenase
LA: Lactic acid
GLU: Glucose
HG: Liver glycogen
SOD: Superoxide dismutase
MDA: Malondialdehyde
TCM: Traditional Chinese Medicine
Nrf2: Nuclear factor erythroid 2-related factor-2
HO-1: Heme oxygenase 1
Keap1: Kelch-like ECH-associated protein 1
PI3K: Phosphoinositide 3-kinase
AKT: Protein kinase B
PPI: Protein and protein interaction
GO: Gene Ontology
KEGG: Kyoto Encyclopedia of Genes and Genomes
NQO1: NAD(P)H quinone oxidoreductase 1
