# Correction: Ren-Shen-Bu-Qi decoction alleviates exercise fatigue through activating PI3K/AKT/Nrf2 pathway in mice

**DOI:** 10.1186/s13020-025-01095-0

**Published:** 2025-04-17

**Authors:** Yangyang Chen, Tinghui Gao, Jing Bai, Wenjing Zhang, Yutong Zhou, Ruichang Zhao, Youhui Deng, Xiaogang Liu, Zhangjun Huang, Songtao Wang, Caihong Shen, Sijing Liu, Jinlin Guo

**Affiliations:** 1 State Key Laboratory of Southwestern Chinese Medicine Resources, College of Pharmacy, Chengdu University of Traditional Chinese Medicine, Chengdu, 611137 China; 2 College of Medical Technology, Chengdu University of Traditional Chinese Medicine, Chengdu, 611137 China; 3 Luzhou Laojiao Group Co. Ltd, Luzhou, People’s Republic of China; 4 National Engineering Research Center of Solid-State Brewing, Luzhou, People’s Republic of China

**Correction: Chinese Medicine (2024) 19:154** 10.1186/s13020-024-01027-4

Following publication of the original article [1], the authors identified errors in Fig. 6G. In detail, the wrong images were used in the p-PI3K in Fig. 6G. The errors were caused by a mistake in the layout and selection of representative images.

The correct abstract figure, Fig. 6 and supplementary figures in Supplementary file 5 have been provided in this Correction.

The incorrect Graphical Abstract is:
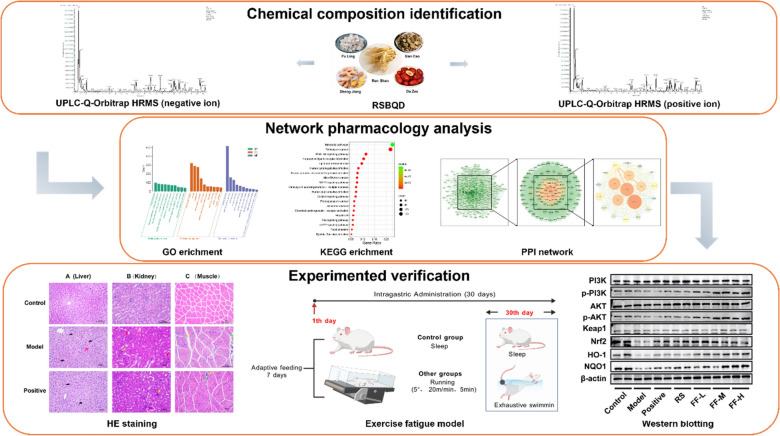


The correct Graphical Abstract is:
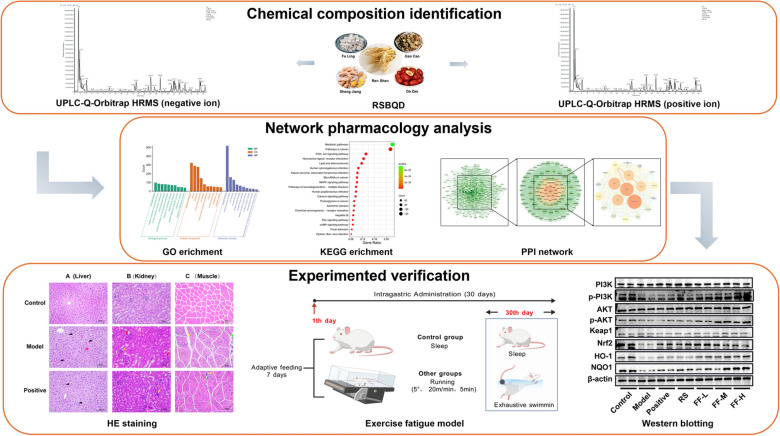


The incorrect Fig. 6 is:

**Figure:**
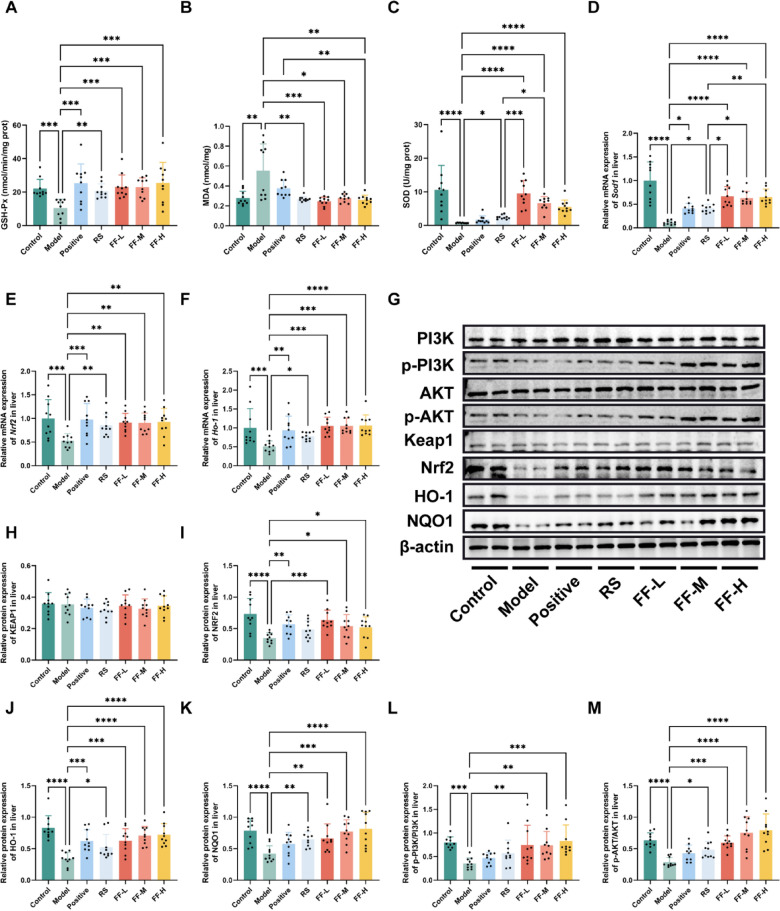
**Fig. 6** RSBQD activated PI3K/AKT/Nrf2 signaling pathway in exercise fatigued mice (n = 10). **A** Hepatic GSH-Px activity. **B** Hepatic SOD activity. **C** Hepatic MDA content. **D–F** The relative mRNA levels of Sod1 (**D**), Nrf2 (**E**), and Ho-1 (**F**). **G** Representative images of Western blot. **H–K** Relative protein levels of KEAP1 (**H**), NRF2 (**I**), HO-1 (**J**), and NQO1 (**K**). (**L**, **M**) The ratio of p-PI3K/PI3K L and p-AKT/AKT **M**. *P < 0.05, **P < 0.01, ***P < 0.001,****P < 0.0001

The correct Fig. 6 is:

**Fig. 6:**
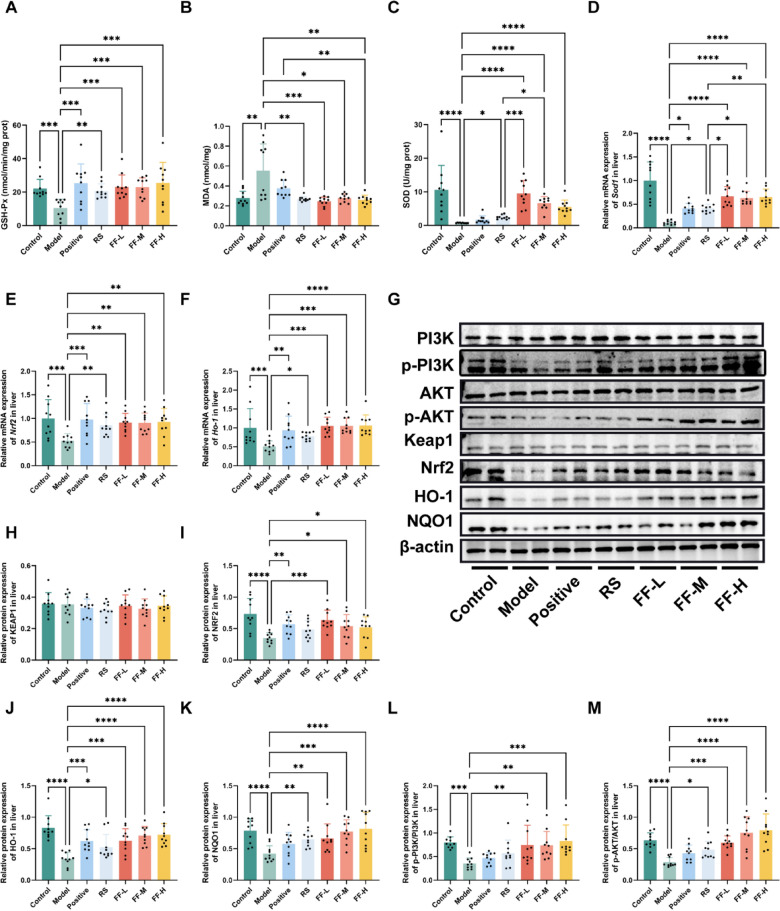
RSBQD activated PI3K/AKT/Nrf2 signaling pathway in exercise fatigued mice (n = 10). **A** Hepatic GSH-Px activity. **B** Hepatic SOD activity. **C** Hepatic MDA content. **D–F** The relative mRNA levels of Sod1 (**D**), Nrf2 (**E**), and Ho-1 (**F**). **G** Representative images of Western blot. **H–K** Relative protein levels of KEAP1 (**H**), NRF2 (**I**), HO-1 (**J**), and NQO1 (**K**). (**L**, **M**) The ratio of p-PI3K/PI3K L and p-AKT/AKT **M**. *P < 0.05, **P < 0.01, ***P < 0.001,****P < 0.0001

The incorrect Supplementary file 5 is:

Supplementary Figures

**Figure:**
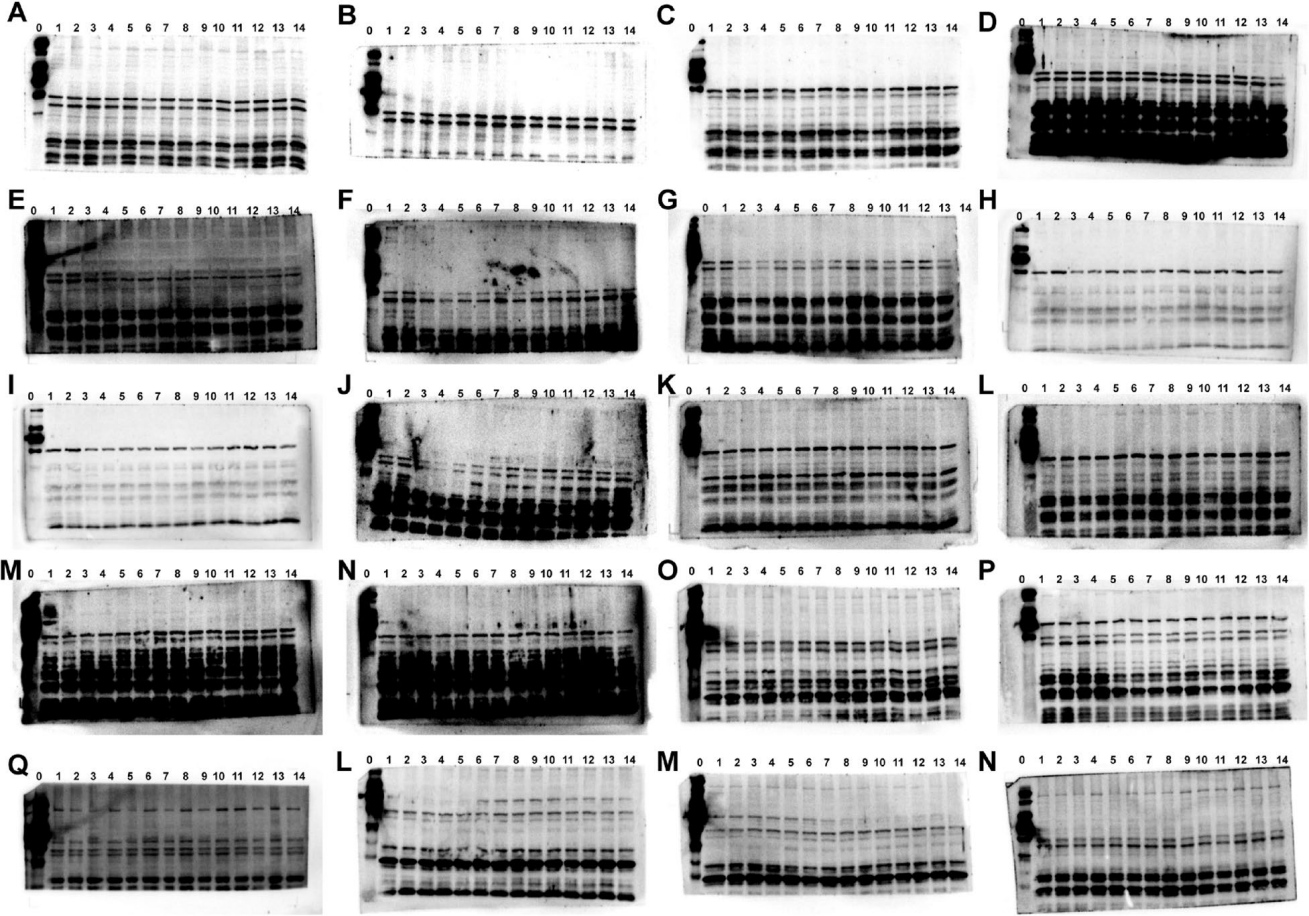
**Fig. S1** Images of the Western blots in fatigued mice. The protein of PI3K (**A-E**), p-PI3K (**F-J**), AKT (**K–O**), and p-AKT (**P-N**). The protein in fatigued mice from control group (No.1–2), model group (No.3–4), positive group (No.5–6), ren shen group (No.7–8), RSBQD low dose group (No.9–10), RSBQD medium dose group (No.11–12), RSBQD high dose group (No.13–14), and the protein ladder (No.0)

**Figure:**
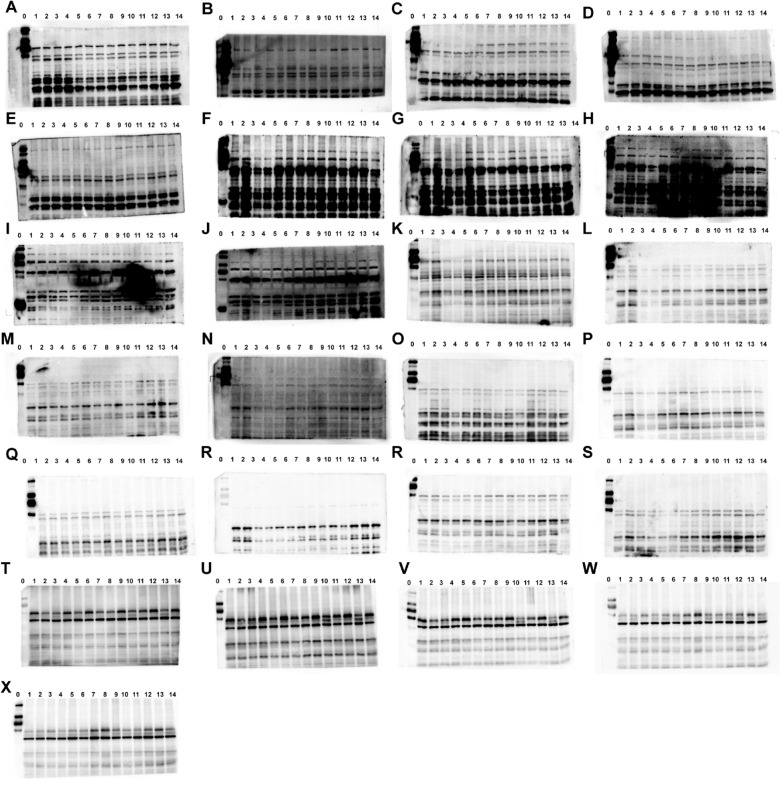
**Fig. S2** Images of the Western blots in fatigued mice. The protein of KEAP1 (**A-E**), NRF2 (**F-J**), HO-1 (**K–O**), NQO1 (**P-S**) and β-actin(**T-X**). The protein in fatigued mice from control group (No.1–2), model group (No.3–4), positive group (No.5–6), ren shen group (No.7–8), RSBQD low dose group (No.9–10), RSBQD medium dose group (No.11–12), RSBQD high dose group (No.13–14), and the protein ladder (No.0)

The correct Supplementary file 5 is:

Supplementary Fig.S1

**Figure:**
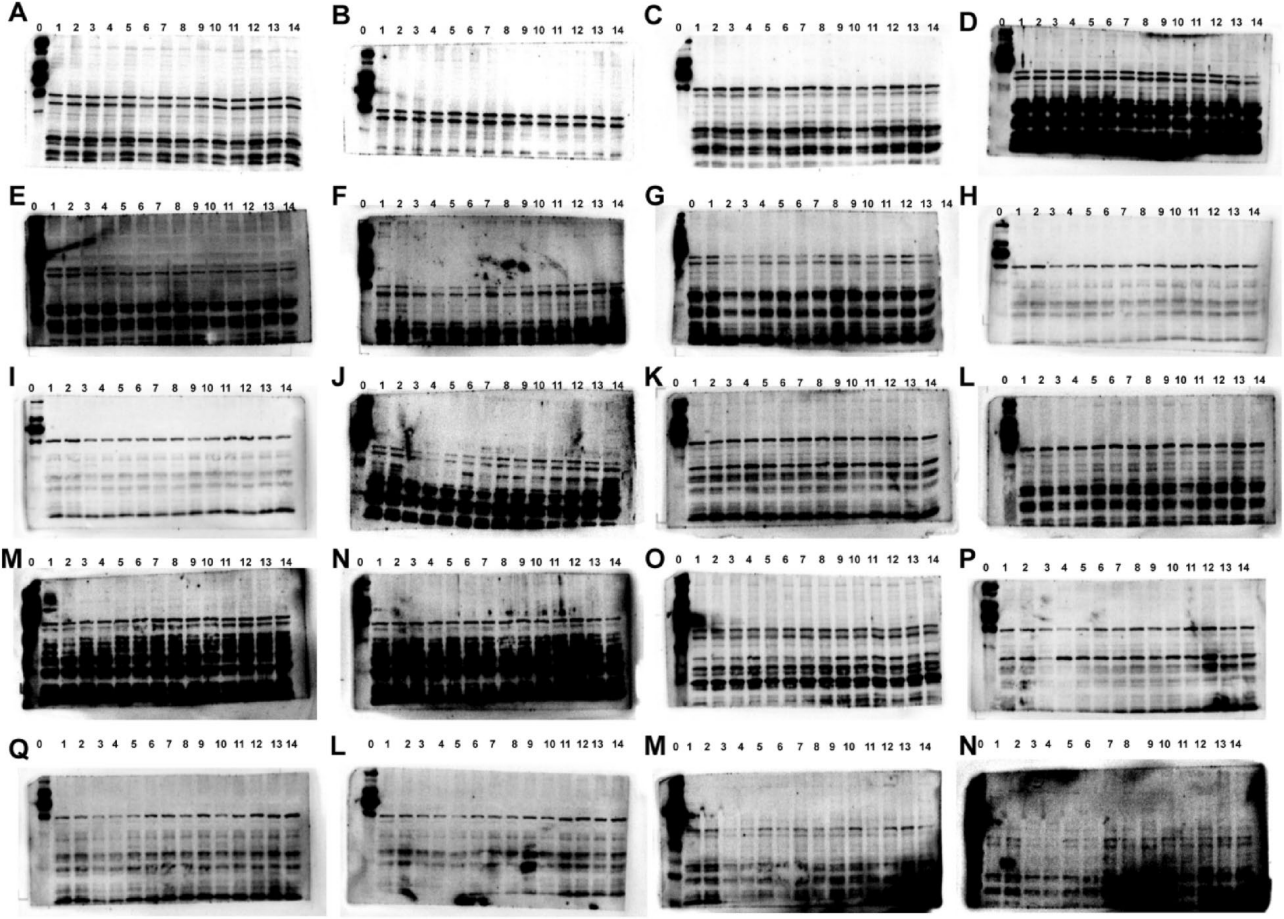
**Fig. S1** Images of the Western blots in fatigued mice. The protein of PI3K (**A-E**), p-PI3K (**F-J**), AKT (**K–O**), and p-AKT (**P-N**). The protein in fatigued mice from control group (No.1–2), model group (No.3–4), positive group (No.5–6), ren shen group (No.7–8), RSBQD low dose group (No.9–10), RSBQD medium dose group (No.11–12), RSBQD high dose group (No.13–14), and the protein ladder (No.0)

Supplementary Fig.S2

**Figure:**
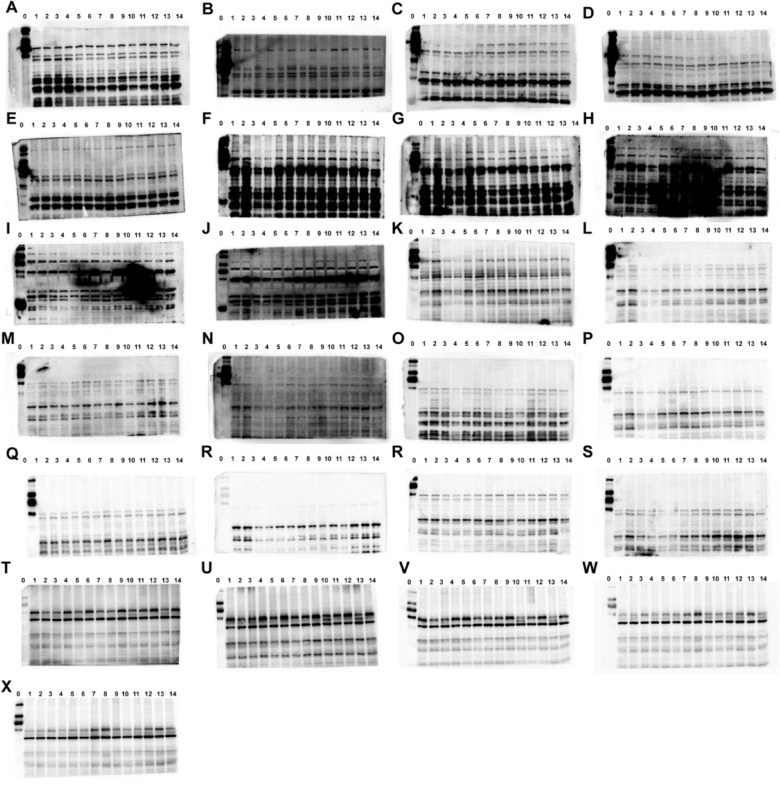
**Fig. S2** Images of the Western blots in fatigued mice. The protein of KEAP1 (**A-E**), NRF2 (**F-J**), HO-1 (**K–O**), NQO1 (**P-S**) and β-actin(**T-X**). The protein in fatigued mice from control group (No.1–2), model group (No.3–4), positive group (No.5–6), ren shen group (No.7–8), RSBQD low dose group (No.9–10), RSBQD medium dose group (No.11–12), RSBQD high dose group (No.13–14), and the protein ladder (No.0)

The authors apologize for the errors and state that this does not change the results and the scientific conclusions of this study. The original article [1] has been corrected.
