# Supplementary material for: Ren-Shen-Bu-Qi decoction alleviates exercise fatigue through activating PI3K/AKT/Nrf2 pathway in mice
Source: Chin Med. 2024 Nov 5;19:154. doi: 10.1186/s13020-024-01027-4 (PMC11539552; doi:10.1186/s13020-024-01027-4)
Supplement: Supplementary file 1 [file 13020_2024_1027_MOESM1_ESM.docx]

**Supplementary Materials and Methods**

*2.3 UPLC-Q-Orbitrap HRMS analysis*

As decribed previously ^[15]^, the chemical composition of RSBQD was analyzed using a Vanquish type UPLC-Q-Orbitrap HRMS and an Thermo Scientific Accucore^TM^ C_18_ column (3 mm × 100mm, 2.6 μm) with a mobile phase of 0.1% formic acid-water (A)-0.1% formic acid-acetonitrile (B) and a linear gradient of 0-6 min (5% B), 6-8 min (5-10% B), 8-12 min (10% B), 12-25 min (10-20% B), 25-35 min (20-40% B), 35-40 min (40% B), 40-45min (40-95% B). The column temperature was set at 25°C, with an injection volume of 10 μL and a flow rate of 0.3 mL/min.

Mass spectrometry analysis was conducted using an electrospray ionization source (ESI) in both positive and negative ion modes. The scanning mode utilized was Full MS/data-dependent secondary scanning (Full MS/dd-MS2), with a m/z scanning range of 100-1500. It operated at a primary resolution of 70,000 and a secondary resolution of 17,500. The ionization spray voltages were set at 3.5 kV (ESI^+^) and 3.0 kV (ESI^-^), and the ion source temperature was set to 350 °C, with a sheath gas flow rate of 35 L/min and an auxiliary gas flow rate of 10 L/min. The ion transfer tube temperature was maintained at 320 °C. Collision energy gradients of 20, 40, and 60 eV were used.

Data analysis was conducted with Compound Discoverer 3.0, utilizing a local high-resolution database of Chinese herbal medicine components and the mzCloud network database. Matching parameters included a peak area threshold of 80,000, a mass deviation of less than 5 ppm for primary quasi-molecular ions and secondary fragments, and a match score above 85. For further compound identification, additional analyses were carried out with Xcalibur, Mass Frontier software, literature references, and databases like Pubchem, and HMDB.

*2.9 RT-qPCR and WB analysis*

Total RNA was extracted using the RNA-easy Isolation Reagent kit (Nanjing Vazyme Bio-technology Co.). The total RNA underwent reverse transcription to cDNA employing the ExonScript® RT SuperMix with dsDNase kit (Nanjing Vazyme Bio-technology Co.). *Nrf2*, *Ho-1*, and *Sod-1* expression was measured. Amplification levels of the target genes relative to controls were quantified using the 2^-ΔΔCt^ method, with *Gapdh* serving as an endogenous reference for data normalization. Table S2 contains primer sequence data for the genes examined.

Total protein extraction from liver tissue was carried out using RIPA lysis buffer (Shanghai Beyotime Biotechnology Co.), 1% phosphorylated protease inhibitor, and 1% PMSF (100 mM). Protein sample concentrations were measured using Mei5 Biotech's BCA protein quantification kit. Subsequently, the protein samples underwent electrophoretic separation on an SDS-PAGE gel followed by transfer to a PVDF membrane. Following the blocking of non-specific binding sites, specific primary and secondary antibodies were applied to the PVDF membranes. The results obtained from chemiluminescence detection were then analyzed using ImageJ software.
