# Supplementary material for: Ren-Shen-Bu-Qi decoction alleviates exercise fatigue through activating PI3K/AKT/Nrf2 pathway in mice
Source: Chin Med. 2024 Nov 5;19:154. doi: 10.1186/s13020-024-01027-4 (PMC11539552; doi:10.1186/s13020-024-01027-4)
Supplement: Supplementary file 2 [file 13020_2024_1027_MOESM2_ESM.docx]

**Table S1. Identification of chemical components of RSBQD by UPLC-Q-Orbitrap-HRMS.**

| **NO** | | **Compounds** | **Formula** | **m/z** | **Reference Ion** | **RT [min]** | | **Area** | **Class** | |
| --- | --- | --- | --- | --- | --- | --- | --- | --- | --- | --- |
| 1 | | Isoliquiritigenin | C15 H12 O4 | 257.08 | [M+H]+1 | 21.03 | 4.39E+09 | | j | |
| 2 | | Naringin | C27 H32 O14 | 579.17 | [M-H]-1 | 25.19 | 3.10E+09 | | a | |
| 3 | | 20(R)-Ginsenoside Rg2 | C42 H72 O13 | 829.50 | [M+FA-H]-1 | 34.37 | 2.40E+09 | | f | |
| 4 | | DL-Norleucine | C6 H13 N O2 | 132.10 | [M+H]+1 | 2.01 | 1.57E+09 | | b | |
| 5 | | Sinapine | C16 H23 N O5 | 310.16 | [M+H]+1 | 11.84 | 1.56E+09 | | e | |
| 6 | | Hesperetin | C16 H14 O6 | 301.07 | [M-H]-1 | 33.64 | 1.48E+09 | | a | |
| 7 | | 3-O-Feruloylquinic acid | C17 H20 O9 | 367.10 | [M-H]-1 | 15.45 | 1.25E+09 | | c | |
| 8 | | Guanosine | C10 H13 N5 O5 | 284.10 | [M+H]+1 | 1.91 | 1.02E+09 | | j | |
| 9 | | Azelaic acid | C9 H16 O4 | 187.10 | [M-H]-1 | 24.91 | 8.91E+08 | | d | |
| 10 | | trans-3-Indoleacrylic acid | C11 H9 N O2 | 188.07 | [M+H]+1 | 6.24 | 8.19E+08 | | j | |
| 11 | | 3-p-coumaroylquinic acid | C16 H18 O8 | 337.09 | [M-H]-1 | 12.97 | 7.89E+08 | | c | |
| 12 | | Zapotin | C19 H18 O6 | 343.12 | [M+H]+1 | 38.10 | 7.21E+08 | | a | |
| 13 | | Poncirin | C28 H34 O14 | 639.19 | [M+FA-H]-1 | 30.78 | 6.15E+08 | | a | |
| 14 | | Kojic acid | C6 H6 O4 | 143.03 | [M+H]+1 | 5.05 | 5.71E+08 | | j | |
| 15 | | Formononetin | C16 H12 O4 | 269.08 | [M+H]+1 | 35.44 | 5.30E+08 | | h | |
| 16 | | Pyrogallol | C6 H6 O3 | 127.04 | [M+H]+1 | 1.77 | 5.29E+08 | | g | |
| 17 | | L-Isoleucine | C6 H13 N O2 | 132.10 | [M+H]+1 | 1.89 | 5.13E+08 | | b | |
| 18 | | Chlorogenic acid | C16 H18 O9 | 353.09 | [M-H]-1 | 10.16 | 4.97E+08 | | c | |
| 19 | | Ginsenoside Rd | C48 H82 O18 | 991.55 | [M+FA-H]-1 | 36.34 | 4.50E+08 | | f | |
| 20 | | Adenosine | C10 H13 N5 O4 | 268.10 | [M+H]+1 | 1.77 | 4.45E+08 | | j | |
| 21 | | 7,8-Dihydroxy-4-methylcoumarin | C10 H8 O4 | 193.05 | [M+H]+1 | 26.50 | 3.31E+08 | | i | |
| 22 | | Semilicoisoflavone B | C20 H16 O6 | 353.10 | [M+H]+1 | 44.28 | 2.75E+08 | | h | |
| 23 | | Caffeic acid | C9 H8 O4 | 179.03 | [M-H]-1 | 10.93 | 2.48E+08 | | e | |
| 24 | | Tangeritin | C20 H20 O7 | 373.13 | [M+H]+1 | 40.80 | 2.45E+08 | | a | |
| 25 | | Cyclo(isoleucylprolyl) | C11 H18 N2 O2 | 211.14 | [M+H]+1 | 15.47 | 2.13E+08 | | b | |
| 26 | | Barpisoflavone A | C16 H12 O6 | 301.07 | [M+H]+1 | 24.88 | 1.90E+08 | | h | |
| 27 | | 4-Coumaric acid | C9 H8 O3 | 165.05 | [M+H]+1 | 15.41 | 1.74E+08 | | e | |
| 28 | | Licoagroside B | C18 H24 O12 | 433.13 | [M+H]+1 | 12.03 | 1.65E+08 | | j | |
| 29 | | Licochalcone A | C21 H22 O4 | 339.16 | [M+H]+1 | 43.18 | 1.65E+08 | | j | |
| 30 | | Corymboside | C26 H28 O14 | 565.16 | [M+H]+1 | 18.61 | 1.62E+08 | | a | |
| 31 | | Ferulic acid | C10 H10 O4 | 195.07 | [M+H]+1 | 18.27 | 1.59E+08 | | e | |
| 32 | | Phloroglucinol | C6 H6 O3 | 127.04 | [M+H]+1 | 34.50 | 1.34E+08 | | g | |
| 33 | | 4-Hydroxybenzaldehyde | C7 H6 O2 | 123.04 | [M+H]+1 | 10.77 | 1.31E+08 | | c | |
| 34 | | D-(-)-Quinic acid | C7 H12 O6 | 191.06 | [M-H]-1 | 12.97 | 1.15E+08 | | e | |
| 35 | | Liquiritin | C21 H22 O9 | 419.13 | [M+H]+1 | 21.02 | 1.13E+08 | | a | |
| 36 | | 4-Indolecarbaldehyde | C9 H7 N O | 146.06 | [M+H]+1 | 6.23 | 1.01E+08 | | j | |
| 37 | | Astragalin | C21 H20 O11 | 447.09 | [M-H]-1 | 24.70 | 1.01E+08 | | a | |
| 38 | | Suberic acid | C8 H14 O4 | 173.08 | [M-H]-1 | 17.93 | 9.84E+07 | | d | |
| 39 | | Afalanine | C11 H13 N O3 | 206.08 | [M-H]-1 | 16.71 | 9.23E+07 | | b | |
| 40 | | Quinic acid | C7 H12 O6 | 191.06 | [M-H]-1 | 15.46 | 8.92E+07 | | c | |
| 41 | | Salicylic acid | C7 H6 O3 | 137.02 | [M-H]-1 | 22.60 | 8.32E+07 | | j | |
| 42 | | Dodecyl sulfate | C12 H26 O4 S | 265.15 | [M-H]-1 | 44.88 | 7.89E+07 | | d | |
| 43 | | Umbelliferone | C9 H6 O3 | 163.04 | [M+H]+1 | 34.37 | 7.82E+07 | | i | |
| 44 | | Scoparone | C11 H10 O4 | 207.07 | [M+H]+1 | 24.39 | 6.90E+07 | | i | |
| 45 | | L-Phenylalanine | C9 H11 N O2 | 164.07 | [M-H]-1 | 2.87 | 6.36E+07 | | b | |
| 46 | | Riboflavin | C17 H20 N4 O6 | 377.15 | [M+H]+1 | 14.30 | 6.30E+07 | | j | |
| 47 | | Abscisic acid | C15 H20 O4 | 263.13 | [M-H]-1 | 29.18 | 6.09E+07 | | f | |
| 48 | | Naringenin | C15 H12 O5 | 271.06 | [M-H]-1 | 25.02 | 6.06E+07 | | a | |
| 49 | | Arctigenin | C21 H24 O6 | 373.16 | [M+H]+1 | 30.01 | 5.69E+07 | | j | |
| 50 | | Tyrosine | C9 H11 N O3 | 182.08 | [M+H]+1 | 18.31 | 5.24E+07 | | b | |
| 51 | | Protopanaxdiol | C30 H52 O3 | 461.40 | [M+H]+1 | 30.88 | 5.15E+07 | | f | |
| 52 | | Rutin | C27 H30 O16 | 609.15 | [M-H]-1 | 21.30 | 5.01E+07 | | a | |
| 53 | | Morin | C15 H10 O7 | 303.05 | [M+H]+1 | 24.70 | 4.92E+07 | | a | |
| 54 | | Oroxylin A | C16 H12 O5 | 283.06 | [M-H]-1 | 33.30 | 4.90E+07 | | a | |
| 55 | | Daidzein | C15 H10 O4 | 253.05 | [M-H]-1 | 28.20 | 4.57E+07 | | h | |
| 56 | | N-Acetyl-DL-tryptophan | C13 H14 N2 O3 | 245.09 | [M-H]-1 | 20.38 | 4.48E+07 | | b | |
| 57 | | Pantothenic acid | C9 H17 N O5 | 220.12 | [M+H]+1 | 4.45 | 4.47E+07 | | c | |
| 58 | | Sinapinic acid | C11 H12 O5 | 225.08 | [M+H]+1 | 18.91 | 4.34E+07 | | e | |
| 59 | | Methyl cinnamate | C10 H10 O2 | 163.08 | [M+H]+1 | 9.35 | 4.25E+07 | | e | |
| 60 | | Eriodictioside | C27 H32 O15 | 595.17 | [M-H]-1 | 22.18 | 4.18E+07 | | j | |
| 61 | | Diosmin | C28 H32 O15 | 609.18 | [M+H]+1 | 25.93 | 4.06E+07 | | a | |
| 62 | | Cyclo(-Pro-Thr) | C9 H14 N2 O3 | 199.11 | [M+H]+1 | 2.46 | 3.86E+07 | | b | |
| 63 | | Dihydrocapsaicin | C18 H29 N O3 | 308.22 | [M+H]+1 | 30.98 | 3.85E+07 | | g | |
| 64 | | Neochlorogenic acid | C16 H18 O9 | 353.09 | [M-H]-1 | 6.03 | 3.77E+07 | | c | |
| 65 | | Catechin | C15 H14 O6 | 289.07 | [M-H]-1 | 13.67 | 3.63E+07 | | a | |
| 66 | | 8-Hydroxyquinoline | C9 H7 N O | 146.06 | [M+H]+1 | 23.45 | 3.61E+07 | | j | |
| 67 | | Apigetrin | C21 H20 O10 | 433.11 | [M+H]+1 | 25.37 | 3.51E+07 | | a | |
| 68 | | 2-Methylglutaric acid | C6 H10 O4 | 145.05 | [M-H]-1 | 2.72 | 3.14E+07 | | d | |
| 69 | | Luteolin | C15 H10 O6 | 285.04 | [M-H]-1 | 30.53 | 3.07E+07 | | a | |
| 70 | | Emodin | C15 H10 O5 | 271.06 | [M+H]+1 | 24.90 | 2.95E+07 | | j | |
| 71 | | Vanillin | C8 H8 O3 | 151.04 | [M-H]-1 | 13.68 | 2.84E+07 | | g | |
| 72 | | Pimelic acid | C7 H12 O4 | 159.07 | [M-H]-1 | 10.84 | 2.76E+07 | | d | |
| 73 | | 6-Methoxyquinoline | C10 H9 N O | 160.08 | [M+H]+1 | 23.45 | 2.72E+07 | | j | |
| 74 | | Rhoifolin | C27 H30 O14 | 579.17 | [M+H]+1 | 25.48 | 2.71E+07 | | a | |
| 75 | | Hispidulin | C16 H12 O6 | 301.07 | [M+H]+1 | 33.30 | 2.60E+07 | | a | |
| 76 | | Kaempferide | C16 H12 O6 | 299.06 | [M-H]-1 | 30.23 | 2.59E+07 | | a | |
| 77 | | Liquiritigenin | C15 H12 O4 | 257.08 | [M+H]+1 | 30.43 | 2.59E+07 | | a | |
| 78 | | (+/-)12(13)-DiHOME | C18 H34 O4 | 313.24 | [M-H]-1 | 43.37 | 2.54E+07 | | d | |
| 79 | | Biochanin A | C16 H12 O5 | 285.08 | [M+H]+1 | 30.95 | 2.54E+07 | | h | |
| 80 | | Vitexin | C21 H20 O10 | 433.11 | [M+H]+1 | 20.96 | 2.14E+07 | | a | |
| 81 | | Ginsenoside Re | C48 H82 O18 | 991.55 | [M+FA-H]-1 | 41.30 | 2.11E+07 | | f | |
| 82 | | Astilbin | C21 H22 O11 | 449.11 | [M-H]-1 | 21.39 | 1.95E+07 | | a | |
| 83 | | 4-Nitrocatechol | C6 H5 N O4 | 154.01 | [M-H]-1 | 14.52 | 1.77E+07 | | g | |
| 84 | | Cynaroside | C21 H20 O11 | 447.09 | [M-H]-1 | 22.32 | 1.73E+07 | | f | |
| 85 | | Quercetin | C15 H10 O7 | 301.04 | [M-H]-1 | 30.63 | 1.72E+07 | | a | |
| 86 | | Asiatic acid | C30 H48 O5 | 487.34 | [M-H]-1 | 42.72 | 1.43E+07 | | f | |
| 87 | | 4,5-Dicaffeoylquinic acid | C25 H24 O12 | 515.12 | [M-H]-1 | 26.80 | 1.02E+07 | | c | |
|  | | | | | | | | |  |  |
| 88 | | 19R-hydroxy-PGF2alpha | C20 H34 O6 | 369.23 | [M-H]-1 | 20.15 | 9.48E+06 | | d | |

a: flavonoids, b: carboxylic acids and derivatives, c: organooxygen compounds, d: fatty Acyls, e: cinnamic acids and derivatives, f: prenol lipids, g: phenols, h: isoflavonoids, i: coumarins and derivatives, j: others
