# Supplementary material for: Ren-Shen-Bu-Qi decoction alleviates exercise fatigue through activating PI3K/AKT/Nrf2 pathway in mice
Source: Chin Med. 2024 Nov 5;19:154. doi: 10.1186/s13020-024-01027-4 (PMC11539552; doi:10.1186/s13020-024-01027-4)
Supplement: Supplementary file 3 [file 13020_2024_1027_MOESM3_ESM.docx]

**Table S2. Sequences of primers used in this study.**

| **Gene/Taxa** | **Forward** **primer sequences (5’→3’)** | **Reverse** **primer sequences (5’→3’)** |
| --- | --- | --- |
| Mus*-Nrf2* | AAGCACAGCCAGCACATTCTCC | TGACCAGGACTCACGGGAACTTC |
| Mus*-Ho-1* | CTCTGACGAAGTGACGCCATCTG | GGACATGAACGTCATTCTCT |
| Mus*-Sod1* | GGGAAGCATGGCGATGAAAG | CCCCATACTGATGGACGTGG |
| Mus-*Gapdh* | TGTGTCCGTCGTGGATCTGA | GATGCCTGCTTCACCACCTT |
