# Supplementary material for: Ren-Shen-Bu-Qi decoction alleviates exercise fatigue through activating PI3K/AKT/Nrf2 pathway in mice
Source: Chin Med. 2024 Nov 5;19:154. doi: 10.1186/s13020-024-01027-4 (PMC11539552; doi:10.1186/s13020-024-01027-4)
Supplement: Supplementary file 4 [file 13020_2024_1027_MOESM4_ESM.docx]

**Table S3. Running training pre-experiment (n=4)**

| **NO** | **Velocity (m/min)** | **Slope(°)** | **Fatigue（min）^a^** | **Exhaustion (min)^b^** |
| --- | --- | --- | --- | --- |
| 1 | 15 | 0 | 30.00±1.06 | 40.12±1.06 |
| 2 | 20 | 0 | 19.93±0.59 | 30.30±0.99 |
| 3 | 25 | 0 | 15.16±0.77 | 25.28±1.07 |
| 4 | 25 | 5 | 4.97±0.16 | 8.09±0.14 |

a: The mice demonstrated a diminished capacity to sustain their running speed despite the application of electrical stimulation. b: The mice exhibited a refusal to continue running despite electrical stimulation, accompanied by severe panting.
