# Supplementary material for: Ren-Shen-Bu-Qi decoction alleviates exercise fatigue through activating PI3K/AKT/Nrf2 pathway in mice
Source: Chin Med. 2024 Nov 5;19:154. doi: 10.1186/s13020-024-01027-4 (PMC11539552; doi:10.1186/s13020-024-01027-4)
Supplement: Supplementary file 5 [file 13020_2024_1027_MOESM5_ESM.docx]

**Supplementary Fig.S1**


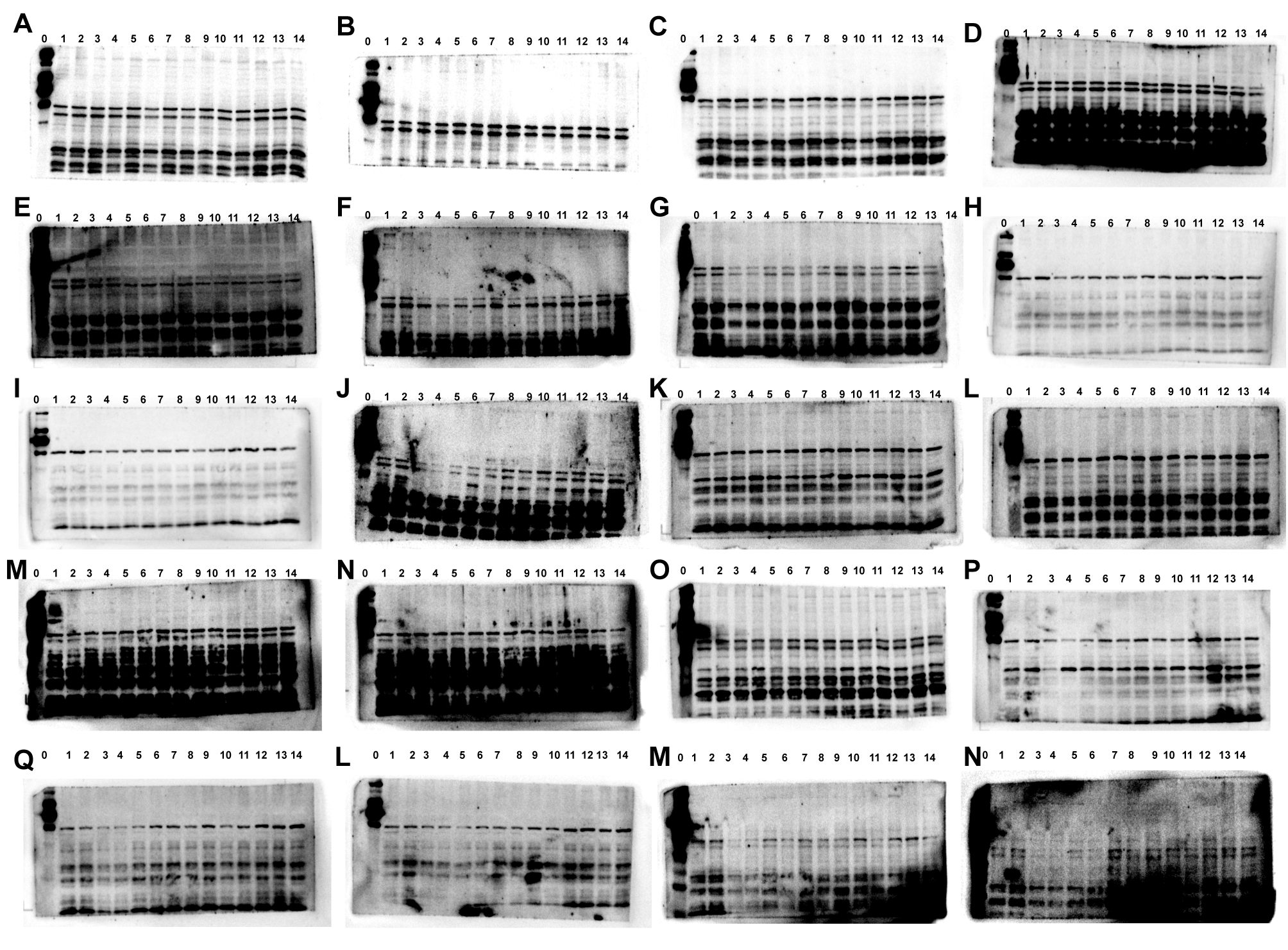


**Fig. S1 Images of the Western blots in fatigued mice.** The protein of PI3K (**A-E**), p-PI3K (**F-J**), AKT (**K-O**), and p-AKT (**P-N**). The protein in fatigued mice from control group (No.1-2), model group (No.3-4), positive group (No.5-6), ren shen group (No.7-8), RSBQD low dose group (No.9-10), RSBQD medium dose group (No.11-12), RSBQD high dose group (No.13-14), and the protein ladder (No.0).

**Supplementary Fig.S2**


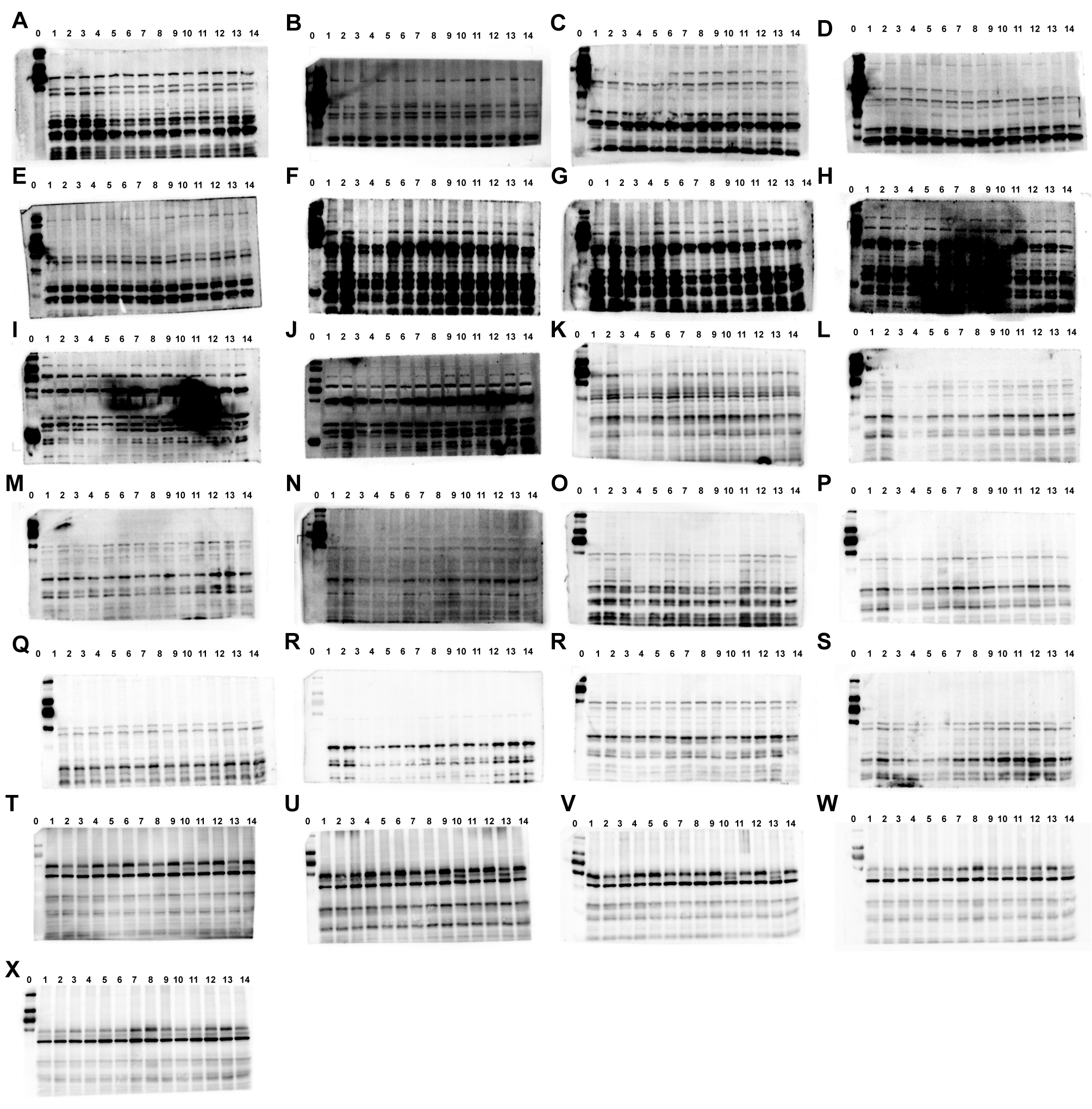


**Fig. S2 Images of the Western blots in fatigued mice.** The protein of KEAP1 (**A-E**), NRF2 (**F-J**), HO-1 (**K-O**), NQO1 (**P-S**) and β-actin(**T-X**). The protein in fatigued mice from control group (No.1-2), model group (No.3-4), positive group (No.5-6), ren shen group (No.7-8), RSBQD low dose group (No.9-10), RSBQD medium dose group (No.11-12), RSBQD high dose group (No.13-14), and the protein ladder (No.0).
